# Supplementary material for: Neuropathic pain caused by miswiring and abnormal end organ targeting
Source: Nature. 2022 May 25;606(7912):137–45. doi: 10.1038/s41586-022-04777-z (PMC9159955; doi:10.1038/s41586-022-04777-z)
Supplement: Supplementary file 1 — This file contains Supplementary Notes 1–4; Supplementary References and legends for Supplementary Videos 1–9 [file 41586_2022_4777_MOESM1_ESM.pdf]

nature portfolio

https://doi.org/10.1038/s41586-022-04777-z

Supplementary information

Neuropathic pain caused by miswiring and abnormal end organ targeting

In the format provided by the authors and unedited

Neuropathic pain caused by mis-wiring and abnormal end organ targeting

Vijayan Gangadharan[1,3], Hongwei Zheng[2], Francisco J. Taberner[1,4], Jonathan Landry[5], Timo Nees[1], Jelena Pistolic[5], Nitin Agarwal[1], Deepitha Männich[1], Vladimir Benes[5], Moritz Helmstädter[3], Björn Ommer[6], Stefan G. Lechner[1], Thomas Kuner[2] and Rohini Kuner[1,*]

Supplementary Information

List of contents:

- **Supplementary video legends** ………………………............ Pages 2-3
- **Supplementary Notes and References** ……….……………..... Pages 4-45
- **Supplementary Note 1: Characterization of mouse lines** …... Pages 4-7
- **Supplementary Note 2: Automated image analysis** ………... Pages 7-35
- **Supplementary Note 3: TH-expressing fibers** ……................. Pages 35-37
- **Supplementary Note 4: Transcriptomics** …………………..…… Pages 37-45

Supplementary video legends

Supplementary video 1: Visualizing nociceptors and tactile fibres in mouse hindpaw digit. Detailed 3D patterns of structure, topographic distribution and termination patterns of YFP-labelled Aß-LTMR fibers in Thy1-YFP mice and mGFP-labelled nociceptors in SNS-mGFP mice derived from multiphoton imaging of a single end phalanx (digit). Repeated imaging sessions over 8-12 weeks in naïve (untreated) mice demonstrating the ability to find and image the same structures across different, independent imaging sessions in vivo, the stability of intensity and patterning of fluorescently labelled fibers as well as the fidelity and accuracy of the registration and segmentation algorithms in representing the fiber patterns in 3D.

Supplementary video 2: Stability of fibres in sham mice over repeated imaging sessions. YFP-labelled Aß-LTMR fibers at baseline and at 42 weeks after sham surgery over the tibial territory in Thy1-YFP mice, showing lack of overt changes in fiber patterning.

Supplementary video 3: Changes in tactile fibres in denervated territory. YFP-labelled Aß-LTMR fibers at baseline and at 42 weeks after SNI surgery over the tibial territory in Thy1-YFP mice, showing lack of recovery of Aß innervation of the denervated tibial digit until this point. The weakly-labelled structures are not fibers (please see Supplementary note 1 for details).

Supplementary video 4: Reinnervation of denervated territory with nociceptors. Recovery GFP-labelled nociceptors at baseline and at 42 weeks after sham surgery over the tibial territory in SNS-mGFP mice, showing lack of overt changes in fiber patterning.

Supplementary video 5: Defective terminal patterning of collaterally sprouted nociceptors. mGFP-labelled nociceptors at baseline and at 42 weeks after SNI surgery over the tibial territory in SNS-GFP mice. Note that intraepidermal free nerve endings of nociceptors are highly reduced at 42 weeks post_SNI. The latter part of the video shows patterning of the original nociceptive fibers forming a mesh-like network in apposition with Meissners at baseline (highlighted in different colors) as well as the

reproduction of these terminations by collaterally sprouting nociceptors post-SNI. Terminations are highlighted with the same false colours across baseline and post-SNI state to facilitate comparisons.

Supplementary video 6: Termination patterns of tactile afferents and nociceptors at Meissner corpuscle zones. Termination patterns of Aß-LTMR fibers in Thy1-YFP mice and nociceptors in SNS-mGFP mice in a single end phalanx (digit) in naïve (untreated) mice, in which the Meissner corpuscle zones are indicated by arrows. One such zone from each of the two reporter mice is shown in higher magnification in Suppl. video 7. Click to see 3D rotation.

Supplementary video 7: High magnification view of termination patterns at Meissner corpuscles. Higher magnification view of innervation of Meissner corpuscles by YFP-labelled Aß-LTMRs in Thy1-YFP mice and termination zones of mGFP-labelled nociceptors in the vicinity of Meissner corpuscles in SNS-mGFP mice. Note that mGFP-labelled nociceptors form fine mesh-like structures covering the top Meissner, and also send small free-endings further above into the epidermis.

Supplementary video 8: Ultrastructural high resolution reconstruction of a Meissner corpuscle innervation in control conditions. High-resolution 3D reconstruction of an entire Meissner corpuscle from the ventral distal phalanx of a control mouse using block-face scanning electron microscopy. Note that the myelinated fibers (false-coloured in green) show a long, winding pattern and are covered by glial cell lamellar wrapping (false-coloured in yellow), connectivity which is key for sensing tactile force. In contrast, although unmyelinated fibers (false-coloured in red) enter the Meissner corpuscle, they do not occupy a large volume and are not covered by lamellae.

Supplementary video 9: Ultrastructural high resolution reconstruction of Meissner corpuscle innervation in neuropathic conditions. High-resolution 3D reconstruction of an entire Meissner corpuscle from the ventral distal phalanx of the tibial territory of a mouse 24 weeks post-SNI using block-face scanning electron microscopy on. Note that in the absence of Aß myelinated fibres (compared to Supplementary video 8), the unmyelinated fibers (false-coloured in red) extensively fill the Meissner volume with a winding trajectory and are aberrantly covered by glial cell lamellar wrapping (false-coloured in yellow).

Supplementary Notes and References

Supplementary Note 1: Detailed characterization of SNS-tdTomato mice and Thy1-YFP mice

SNS-Cre line-based reporter expression:

The SNS-Cre mice were generated by using about 90 Kb of the promoter elements of the mouse Scn10a gene (encoding Nav1.8) in a BAC transgene to drive expression of Cre recombinase (Agarwal et al. 2004). We have characterized the SNS-Cre mouse line extensively in a number of previous studies and showed that about 95% of cells showing Cre recombination in the DRG are Substance P-positive (peptidergic) or IB4-positive (putative non-peptidergic) nociceptors. Although there have been reports over the recent years of Nav1.8 expression in a few other cell types in the periphery, such as keratinocytes, this does not hold true for SNS-Cre mice, likely because unlike genetically engineered knock-in lines, the SNS-Cre line is a transgene and therefore does not include all of the promoter and enhancer elements of the endogenous *Scn10a* gene. Here, in SNS-tdTomato mice, we observed that 46 % of tdTomato-labelled neurons co-expressed Calcitonin gene regulated peptide (CGRP) or Substance P (SP) (putative peptidergic nociceptive cells), and another 50.5% of tdTomato-labelled DRG neurons demonstrated IB4-binding (putative, non-peptidergic nociceptive neurons), consistent with the recombination profile that we have described previously (Agarwal et al. 2004; Gangadharan et al, 2011; Luo et al., 2012). These populations entail both C-nociceptors as well as A-delta nociceptors. A subpopulation of Cre-expressing neurons also express tyrosine hydroxylase (TH), which is known to be expressed in sympathetic neurons and in a small number of non-peptidergic small and medium-diameter neurons of the DRG, which are believed to be largely C-low threshold mechanoceptors (C-LTMRs) that can be activated by light touch. In the murine lumbar DRGs, the percentage of TH-positive neurons has been reported to range from 5-15% across different studies. Here, we found that about 8% of dtTomato-positive neurons were positive for TH, consistent with the C-LTMR population being a subpopulation of C-fiber neurons (Delfini et al., 2013; Usoskin et al., 2015).

Even in pathological states, such as after nerve injury, the reporter expression in SNS-Cre mice is retained and does not appear ectopically in other sensory types. Nevertheless, to control for this potential scenario, we also verified the key results derived from mGFP imaging experiments in SNS-mGFP mice with immunohistochemical staining of endogenous marker proteins that are expressed in specific sensory populations and employed multiple markers to consolidate the data (Fig. 3 and Extended Data Figs. 7, 8 and 9).

Thy1-YFP reporter mice:

There are several transgenic lines expressing YFP under a Thy1 promoter fragment, which typically imparts mosaic expression in several neural structures and outside of the nervous system. Here, we employed a line which expresses YFP in 13% of DRG neurons. At least 95% of YFP-expressing neurons are NF200-positive, indicating that they are myelinated, large diameter neurons. Although 17% of YFP neurons showed co-immunofluorescence for CGRP, there was 0% overlap with SP, another marker of peptidergic nociceptors, 0% overlap with IB4-binding, indicating absence of YFP expression in a majority of peptidergic nociceptors and all of non-peptidergic nociceptors. Consistent with a minor overlap with tdTomato expression in SNS-tdTomato mice (2%), a few thin YFP-positive fibers were seen in deeper parts of the paw skin, which also showed recovery in form of collateral sprouting from 30 weeks onwards post-SNI in the denervated tibial territory (Extended Data Fig. 3). TH-expressing DRG neurons (presumably C-LTMRs) also completely lacked YFP expression. These findings at the level of DRG somata, taken together with the highly distinct morphology of large-diameter YFP-expressing fibers and their terminations at the dermis-epidermis border in the paw skin, indicate that an overwhelming majority of the YFP-positive fibers are Aß-LTMRs. YFP-positive fibers showed the classical pattern of innervation of Meissner corpuscles in the glabrous skin, in which they were found to wind around the S100-positive glial cells that comprise the corpuscle (upper panels in Extended Data Fig. 7c).

Because reporter expression can change in transgenic lines in models of injury, we backed up the data obtained with YFP imaging with immunohistochemical staining of NF200 as an endogenous marker of Aß-LTMRs in mice post-injury (Fig. 3d, Extended

Data Fig. 4a) and also performed electrophysiological recordings to identify Aß-LTMRs functionally in sham and SNI mice (Fig. 3f,g; Extended Data Fig. 10).

Interestingly, we also observed that when YFP-positive Aß-LTMRs degenerate and disappear in the tibial innervation territory within 3 days of ligation and cutting of the tibial nerve in the SNI model, new YFP expressing-structures appeared in the denervated territory. These did not comprise any blebs of nerve that persist following Wallerian degeneration, but rather resembled clear cellular structures with a stellate morphology (Extended Data Fig. 3). A literature search revealed that this morphology is typical to dendritic cells, an immune cell scavenger population that are associated with clearing debris after cell injury (Hsieh and Lin, 1999; Stankovic et al., 1999; Doss and Smith, 2014). At present, the significance of these cells to nerve regeneration and sprouting or nociceptive sensitivity in the context of the present study is unclear, but will be interesting to analyse in future studies.

References:

Agarwal N, Offermanns S, Kuner R. Conditional gene deletion in primary nociceptive neurons of trigeminal ganglia and dorsal root ganglia. Genesis. 2004 Mar;38(3):122-9.

Delfini MC, Mantilleri A, Gaillard S, Hao J, Reynders A, Malapert P, Alonso S, François A, Barrere C, Seal R, Landry M, Eschallier A, Alloui A, Bourinet E, Delmas P, Le Feuvre Y, Moqrich A. TAFA4, a chemokine-like protein, modulates injury-induced mechanical and chemical pain hypersensitivity in mice. Cell Rep. 2013 Oct 31;5(2):378-88.

Doss AL, Smith PG. Langerhans cells regulate cutaneous innervation density and mechanical sensitivity in mouse footpad. Neurosci Lett. 2014 Aug 22;578:55-60.

Gangadharan V, Wang R, Ulzhöfer B, Luo C, Bardoni R, Bali KK, Agarwal N, Tegeder I, Hildebrandt U, Nagy GG, Todd AJ, Ghirri A, Häussler A, Sprengel R, Seeburg PH, MacDermott AB, Lewin GR, Kuner R. Peripheral calcium-permeable AMPA receptors regulate chronic inflammatory pain in mice. J Clin Invest. 2011 Apr;121(4):1608-23.

Hsieh ST, Lin WM. Modulation of keratinocyte proliferation by skin innervation. J Invest Dermatol. 1999 Oct;113(4):579-86.

Luo C, Gangadharan V, Bali KK, Xie RG, Agarwal N, Kurejova M, Tappe-Theodor A, Tegeder I, Feil S, Lewin G, Polgar E, Todd AJ, Schlossmann J, Hofmann F, Liu DL, Hu SJ, Feil R, Kuner T, Kuner R. Presynaptically localized cyclic GMP-dependent protein kinase 1 is a key determinant of spinal synaptic potentiation and pain hypersensitivity. PLoS Biol. 2012;10(3):e1001283.

Stankovic N, Johansson O, Hildebrand C. Increased occurrence of PGP 9.5-immunoreactive epidermal Langerhans cells in rat plantar skin after sciatic nerve injury. Cell Tissue Res. 1999 Nov;298(2):255-60.

Usoskin D, Furlan A, Islam S, Abdo H, Lönnerberg P, Lou D, Hjerling-Leffler J, Haeggström J, Kharchenko O, Kharchenko PV, Linnarsson S, Ernfors P. Unbiased classification of sensory neuron types by large-scale single-cell RNA sequencing. Nat Neurosci. 2015 Jan;18(1):145-53.

Supplementary Note 2: Automated image analysis

The code for all analysis procedures described here is available at https://github.com/tkunerlab/pns-2photon-longitudinal-3Ddata-analysis.

I. Automated stitching of four 16-bit raw image stacks into a superstack

As illustrated in **Supplementary Fig. 1**, a maximum intensity projection (MIP) image is calculated for each stack and SIFT/SURF is applied for pairs of MIPs. Using MIPs was a precondition to automate the procedure, because the border area would always include a sufficient number of structures for finding correspondences. The parameters identified in pair-wise MIP stitching were then applied to the 3D stacks to obtain a 16-bit "superstack" covering the entire end-phalanx. To avoid disturbances by the original image overlap boundary, we applied a stitching algorithm using watershed and graph cut (Gracias, 2009, Boykov et al., 2001, Kolmogorov, 2004) to reduce the noisy influences of original image boundary. In this stitching and blending workflow, pair-wise image blending is performed independently in each region by means of watershed segmentation and graph cut optimization.

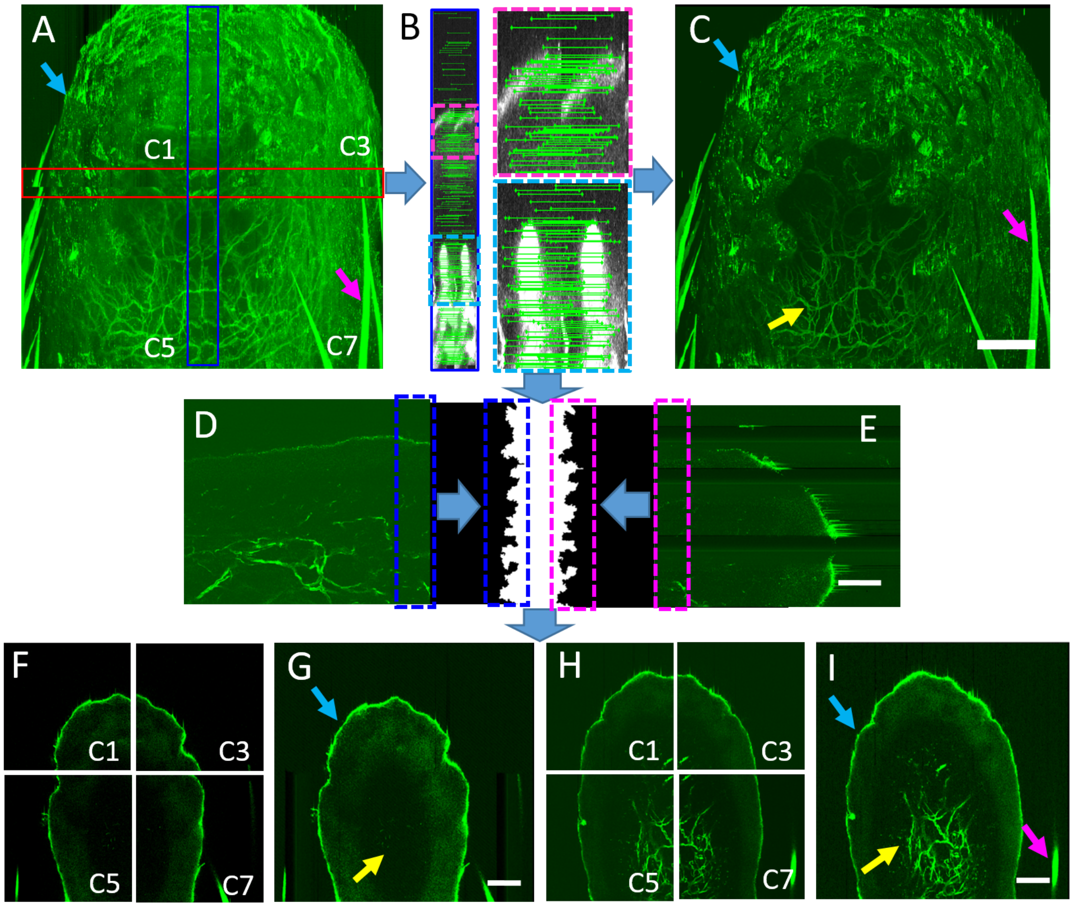

Supplementary Fig. 1 | Automated 3D seamless stitching of four stacks into a superstack. **A,** MIPs of four stacks C1, C3, C5, C7 with overlap region indicated by boxes. First C1 and C3 are stitched, then C5 and C7, then the stitched C1/C3 and C5/C7 MIPs. **B,** Automated correspondence searching in the overlap region. **C,** Stitched MIP image. **D and E,** Correspondence information for image pairs, seamless stitching based on watershed and graph-cut optimization. The black and white binary curves are computed using watershed from the overlap regions marked in blue and magenta boxes. **F to I,** Examples of image frames at a depth of 53 μm (F, G) and 106 μm (H, I). Arrows: epidermis border (blue), neuronal fibers (yellow), and hair (magenta). Scale bar, 100 μm.

II. Segmentation and removal of epidermal autofluorescence via weakly-supervised learning and label propagation

Our workflow removes epidermal autofluorescence and hair, yet retains nerve fibers in each 16-bit superstack. A weakly-supervised regularization approach was developed on discrete graph spaces for perceptual image segmentation. The weakly-supervised

learning combines the labeled information (mask of surrounding epidermal autofluorescence and nerve fibers) and unlabeled data through label propagation for perceptual image segmentation. In this step, each image in a superstack will be segmented according to the segmentation parameters and label information obtained in 3D space. Epidermal irregularities and hair in the 3D image stack can be accurately classified and removed (**Supplementary Fig. 2A-C**).

Top-down and bottom-up segmentation strategy: statistical label propagation

To improve the segmentation results, we added global 3D label information for enhancing the label propagation (Fan et al., 2018, Levin et al., 2008, Zhu et al., 2002) with global constraints in a semantic way. In this approach, a spectral clustering method is embedded and extended into regularization on discrete graph spaces. Thus, te spectral graph clustering is optimized and smoothened by integrating top-down and bottom-up processes via semi-supervised learning. Second, a nonlinear diffusion filter is used to maintain semi-supervised learning, labeling and differences between foreground or background regions. Furthermore, the spectral segmentation is penalized and adjusted using labeling prior and optimal window-based affinity functions in a regularization framework on discrete graph spaces.

Notations and their corresponding descriptions

An image is formulated as a weighted undirected graph $G = (V, E, A)$ with nodes $v \in V$, and edges $e \in E \subseteq V \times V$. Each node v_i represents an image pixel x_i. An edge e_{ij} connects two nodes v_i and v_j in 8-connected neighborhood system. A is the associated affinity matrix. A is symmetric and non-negative. The diagonal matrix $D = diag(D_{11} \dots, D_{nn})$ is called the degree matrix of graph G, where $D_{ii} = \sum_{j=1}^{N} A_{ij}$, Then $L = D - A$ is called the un-normalized graph Laplacian of G. Assuming G is connected (i.e., any node is reachable from any other node), L has certain properties of a connected graph such as symmetric and positive semi-definite, etc.

To remove epidermal autofluorescence and hair, one input image I from a input 3D stack including a foreground image F and a background image B. The intensity of the ith pixel is assumed to be a linear combination of the corresponding foreground and background intensity value,

$$I_i = \alpha_i F_i + (1 - \alpha_i) B_i, \tag{1}$$

where α_i is the pixel's foreground opacity. In this equation, all quantities on the right side of the compositing equation are unknown and some assumptions on the nature of F, B and/or α are needed.

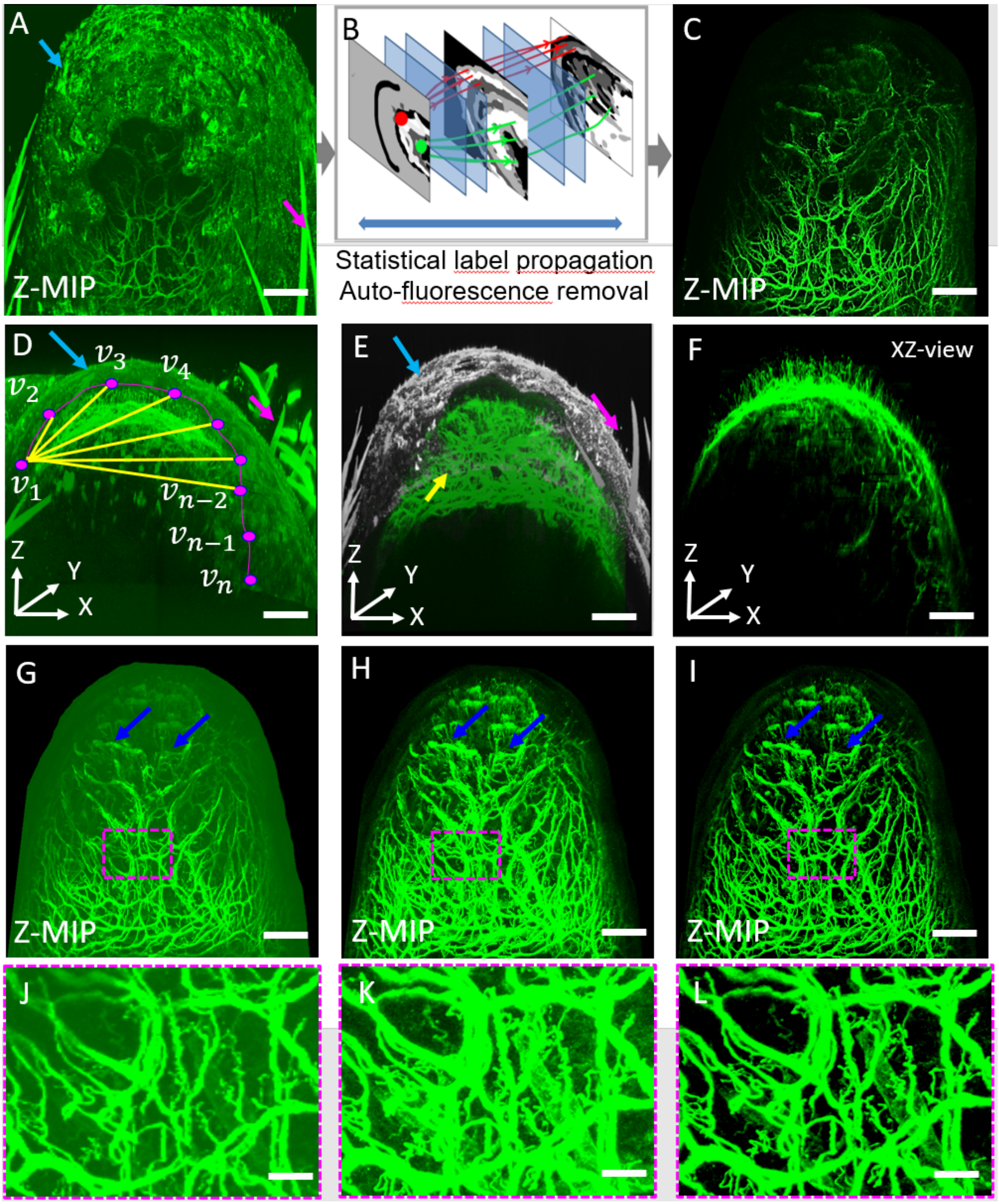

Supplementary Fig. 2 | Removing epidermal autofluorescence and image enhancement of 16-bit 3D image stacks. A, MIP of superstack. **B,** Statistical label propagation workflow. **C,** MIP after applying the algorithm. **D,** Geodesic label propagation in Z-direction on 3D epidermal autofluorescence and appendages. Yellow lines indicate Euclidean distance, magenta

curve indicates geodesic distance, v is vertex node. **E,** Segmented nerve fibers (green) and epidermis/hair (gray). Arrows: epidermis (blue), hair (magenta), nerve fibers (yellow). **F,** Segmented nerve fibers (green) after removal of epidermal autofluorescence and hair. Note that epidermal fibers are fully maintained. **G,** MIP image of 16-bit raw data. **H,** After conversion to 8-bit MIP image using ImageJ function adjust brightness & contrast). **I,** After conversion to 8-bit MIP image using our algorithm (steps 1 and 2). **J-L**, Magnified view of magenta ROI in G-L. Arrows point to Meissner corpuscles. Scale bars 100 μm (A-L), 20 μm (J-L).

Notations and their corresponding descriptions

An image is formulated as a weighted undirected graph $G = (V, E, A)$ with nodes $v \in V$, and edges $e \in E \subseteq V \times V$. Each node v_i represents an image pixel x_i. An edge e_{ij} connects two nodes v_i and v_j in 8-connected neighborhood system. A is the associated affinity matrix. A is symmetric and non-negative. The diagonal matrix $D = diag(D_{11} \dots, D_{nn})$ is called the degree matrix of graph G, where $D_{ii} = \sum_{j=1}^{N} A_{ij}$, Then $L = D - A$ is called the un-normalized graph Laplacian of G. Assuming G is connected (i.e., any node is reachable from any other node), L has certain properties of a connected graph such as symmetric and positive semi-definite, etc.

To remove epidermal autofluorescence and hair, one input image I from a input 3D stack including a foreground image F and a background image B. The intensity of the ith pixel is assumed to be a linear combination of the corresponding foreground and background intensity value,

$$I_i = \alpha_i F_i + (1 - \alpha_i) B_i, \quad (1)$$

where α_i is the pixel's foreground opacity. In this equation, all quantities on the right side of the compositing equation are unknown and some assumptions on the nature of F, B and/or α are needed.

Regularized semi-supervised cost function

To derive our solution for the grayscale case we make the assumption that both F and B are approximately constant over a small window around each pixel. Note that assuming F and B are locally smooth does not mean that the input image I is locally smooth, since discontinuities in α can account for the discontinuities in I. This assumption expresses α as a linear function of the image I, $\alpha_i \approx aI_i + \mathrm{b}, \forall i \in w$,

where $a = \frac{1}{F-B}, b = \frac{B}{F-B}$ and w is a small window. The linear relation is similar to the prior used in (Grady et al., Levin, 2008, 2005, Fan et al., 2018). Our goal is to find a and b minimizing the cost function

$$J(\alpha, a, b) = \sum_{j\in I}(\sum_{i\in w_j}(\alpha_i - a_j I_i - b_j)^2 + \varepsilon a_j^2), \quad (2)$$

where w_j is a small window around pixel j. This cost function has a regularized term on a for numerical stability. This function can be converted as a quadratic cost function in the formula with α unknowns, $J(\alpha) = \alpha^T L\alpha$. Here we can consider a Laplacian diffusion matrix L, which is a symmetrically positive definite matrix.

<u>User label constraints</u>

To achieve reasonable segmentation of epidermal autofluorescence and appendages and internal nerve fibers, biologists mark and label several constraints in image 3D stacks. We mark autofluorescence components in black pixels ($\alpha = 0$) and consider neural fibers as foreground ($\alpha = 1$). Based on the utilization of label constraints, we solve the following equation,

$$\alpha = argmin\, \alpha^T L\alpha + \lambda(\alpha^T - b_S^T)D_S(\alpha - b_S), \quad (3)$$

where λ is chosen as a number for optimizing the segmentation region, D_S is a diagonal matrix whose diagonal elements are one for constrained pixels and zero for all other pixels, and b_S is the vector containing the specified alpha values for the constrained pixels and zero for all other pixels. Since the above cost is quadratic in alpha, the global minimum may be found by differentiation of the equation and setting the derivatives to zero. This amounts to solving the following sparse linear system,

$$(L + \lambda D_S)\alpha = \lambda b_S. \quad (4)$$

In this equation, let I be an image formed from F and B according to the compositing equation (1), and let α^* denote the true segmentation. If F and B satisfy the model in every local window w_k and if the label constraints S are consistent with α^*, then α^* is an optimal segmentation solution for the system (3), where L is constructed with $\epsilon = 0$.

For example, the extracted segmentation region and the smallest eigenvectors tend to be piecewise constant over the same regions. If the values inside a segment in the

eigenvector image are coherent, a single label within such a segment should suffice to propagate the desired value to the entire segment. On the other hand, areas where the eigenvector's values are less coherent correspond to more "difficult" regions in the image, suggesting that more label efforts might be required there.

Spectral Laplacian affinity clustering

The matrix $L = D - A$ is the graph Laplacian for segmentation. The matrix L has the same form as the graph Laplacian used in spectral methods for segmentation but with a novel affinity function given by (Fan et al., 2018). For the typical way to define the affinity function (for example, for image segmentation using normalized cuts (Shi et al, 1997) is to set

$$A_G(i,j) = e^{-\|I_i - I_j\|^2/\sigma^2}, \tag{5}$$

where σ is a global constant. This affinity is large for nearby pixels with similar intensity and approaches zero when the gray value difference is much greater than σ. The transformation is image-dependent and is estimated using a manifold learning technique. Based on the Laplacian as $L = D - W$, we obtain the affinity function with certain constraints provided by label propagated information.

$$A_M(i,j) = \sum_{k(i,j)\in w_k} \frac{1}{|w_k|}\left(1 + (I_i - \mu_k)\left(\Sigma_k + \frac{\epsilon}{|w_k|} I_3\right)^{-1} (I_j - \mu_k)\right). \tag{6}$$

These two affinity functions A_G and A_M can be examined by using eigenvectors as guides for achieving optimal segmentation. The smallest eigenvectors of the Laplacian can guide the user where to label and mark.

Iterative optimization with soft constraints in multi-resolution

The optimization problem defined by equation (3) minimizes a quadratic regularization function subject to linear constraints, and the solution can therefore be found by solving a sparse set of linear equations. Large images or image 3D stacks cannot be solved using Matlab's solver due to memory limitations. To overcome this, we use a coarse-to-fine multi-resolution scheme. We down-sample the image and the constraints and solve it at a lower resolution. The recovered α region is then interpolated to the finer resolution. The α values are thresholded and pixels with α close to zero or one are

clamped and considered as constrained in the finer resolution. Therefore, we minimize the regularization function in the equation

$$\min \sum_{i \in I} (\alpha_i F_i + (1 - \alpha_i) B_i - I_i)^2 + |\alpha_{ix}|((F_{ix})^2 + (B_{ix})^2) + |\alpha_{iy}|((F_{iy})^2 + (B_{iy})^2)$$

where $F_{ix}, F_{iy}, B_{ix}, B_{iy}$, are the x and y derivatives of F and B, and α_{ix} and α_{iy} are the segmentation region derivatives. We note that for a fixed α, this function is quadratic, and its minimum can be found by solving a sparse set of linear equations. Given the solution of F and B, the α solution be further refined according to the practical problems.

For certain complex distribution of phalangeal nerve fibers, epidermal autofluorescence and appendages, such a segmentation-based reconstruction may produce noisy results, and therefore, we solve for F and B using the compositing equation with some explicit soft and hard constraints. Soft constraints include smoothness priors on F and B. The reconstructed image is composed of foreground neural fibers in the central region and background distributed in the surrounding regions. Constrained pixels maybe eliminated from the system, reducing the system size. For that, we note that within the constrained areas, there is no need to enforce the local linear models.

Therefore, when computing the segmentation Laplacian matrix in the equation (4) and (6), we sum only windows w_k that contain at least one unconstrained pixel. Aside from efficiency, clamping alpha values to zero or one is also useful in avoiding an over-smoothed alpha region. We note, however, that clamping the alpha values in a coarse to-fine approach has the adverse effect that long thin structures such as strains of hair may be lost.

Improved 3D segmentation using Z-direction geodesic coherent label propagation in image 3D stacks

Based on our datasets, we needed to achieve maximal accuracy to segment and remove the epidermis with strong intensity, partial epidermis inner buffer region, hair and other autofluorescence. Therefore, stronger prior information (e.g., hard constraints) were combined with soft constraints such as smooth priors for computing an accurate segment boundary between foreground and background.

Our datasets are multiple image 3D stacks. In practice, we give certain sparse labels from the top of the scanning layers and also some labels at the bottom layers in 3D image stacks. The goal of this label information is not only for 2D segmentation between foreground and background in 2D images, but also for utilizing z-direction geodesic coherent information **(Supplementary Fig. 2D-F)**, pairwise similarity and connectivity features. In z-direction following the depth of image 3D stacks, the geodesic metric data similarity is much more effective than previous Euclidean distance-based similarities.

The computation of eigenvectors (Chennubhotla, 2005) of the Laplacian depend only on the input image and are independent of the user's label constraints. It is not necessary to compute them, unless the user wishes to use them for guidance in scribble placement, as described earlier. In this case, they only need to be computed once, possibly as part of the initialization that takes place when a new image is loaded. With geodesic z-direction propagation in image 3D stacks, the coherent pairwise similarities propagate a closing boundary in z-direction from the top layer to the bottom layer including epidermal autofluorescence and appendages.

III. 16-bit to 8-bit conversion of superstacks by nonlinear adaptive depth-dependent adjustment on global and local scales

To facilitate the analysis steps described in sections 4 and 5, the data was converted from 16-bit to 8-bit TIFF files using an approach that maximally preserved the dynamic range of the signal. We proposed a global and local contrast adjustment and combinatorial optimization approach for adaptively adjusting the non-uniform illumination image stacks. The balance of local contrast adjustment and the global contrast adjustment is optimized based on a contrast-brightness based pixel-level weighted fusion framework without losing any relevant information. The optimization framework is formulated in the following,

$$E_I(i) = \widehat{w}_G(i) \cdot G_I(i) + \widehat{w}_L(i) \cdot L_I(i), \quad (1)$$

Where $E_I(i)$ is an optimal enhanced image, i is the index of pixels in a given 16-bit image. We compute the weight maps for the intensity images in a global weight $\widehat{w}_G$

based on a global image intensity $G_I(i)$, and the local weight map $\widehat{w}_L$ based on a local image intensity $L_I(i)$. The result in this equation is an optimal fused image in a Laplacian pyramid method (Burt, et al., 1983).

Step 1: Global adaptive contrast and brightness enhancement for gray images

A general histogram modification framework (Arici, 2009) was used to obtain a mapping function for the optimal modified histogram. We consider a gray image I, with a total number of N pixels and an intensity level range of $[0, R-1]$, R is a 16-bit value 65536. The normalized histogram h_I of the image I is computed in equation (2).

$$h_I(s) = \frac{n_s}{N}, \tag{2}$$

where $s \in [0, R-1]$ is pixel values, and n_s is the total number of pixels having the same value s. The modified histogram h should be closer to the normalized uniform histogram h_U and the value of the residual $h - h_I$ should be small. The problem of obtaining the optimal modified histogram $\tilde{h}$ can be regarded as a solution of a bi-criteria optimization problem, which can be formulated as a weighted sum of two terms as Eq. (3).

$$\min(\|h - h_I\| + \lambda\|h - h_U\|), \tag{3}$$

where $\|h - h_I\|$ is a norm of $h - h_I$, $\|h - h_U\|$ is a norm of $h - h_U$, and the parameter $\lambda > 0$ adjusts the trade-off between the contrast enhancement and the fidelity of the data.

Step 2: Local contrast adaptive adjustment and global optimization framework

Local contrast was enhanced using the framework represented in equation (4) and the Contrast-Limited Adaptive Histogram Equalization method (CLAHE) (Pizer, 1987).

$$G(i) = \begin{cases} \frac{G_I(i)}{I(i)} * \hat{X}(i), & if\ G_I(i)/I(i) \le 0 \\ \frac{R-1-G_I(i)}{R-1-I(i)} * \left[\hat{X}(i) - I(i)\right] + G_I(i), & if\ G_I(i)/I(i) > 1 \end{cases}, \tag{4}$$

Where $I(i)$ is a given image. $\hat{X}(i)$ is an intermediate linear stretching image in the full range of $\hat{X}(i) = (R-1)\frac{X - X_{min}}{X_{max} - X_{min}}$, where X_{max} and X_{min} are the maximal and minimal intensity values in the 16-bit image X. Since the dynamic range of images in each scanning layers are changeable and the acquisition position of mice fingers are

often different, our approach has a robust 16-bit to 8-bit images conversion results based on the measure and adaptively adjustment.

We implemented the function of the proposed method in Matlab and compared it with several state-of-the-art image enhancement algorithms. A comparison with image J brightness & contrast adjustment) illustrates the superior performance of our approach (**Supplementary Fig. 2G-L**).

IV. Automated rigid and non-rigid 4D registration of stacks acquired at different time points

First, automated registration was implemented in a stratified 3D model registration framework, which efficiently handles a hierarchical pyramid multi-resolution 3D image stack. Our algorithms compute the multimodal 3D registration in a stratified approach with respect to the rigid and non-rigid 3D alignment including 3D-to-3D pose correction, 3D-to-3D rigid transformation and 3D-to-3D linear and nonlinear registration (**Supplementary Fig. 3A-D).**

- First, the 3D pose of a given image stack is automatically corrected using the relative 3D pose of the consecutive image stack.
- Second, the rigid linear alignment in the iterative closest points (ICP) (Bergevin, 1996, Besl, 1992) and the nonlinear registration in the thin-plate-splines (TPS) (Bookstein, 1989, Szeliski, 1996, Zheng et al., 2009) are stratified into two steps for reducing and avoiding the over-blending effects in linear and nonlinear 3D registration.
- The results of the stratified matching engines are integrated to achieve higher accuracy of 3D registration of nerve fibers.

Following this strategy, the original global distribution and locally detailed structure of nerve fibers are largely and optimally preserved after 3D registration. The approach performs efficiently in particular for large-scale 3D data alignment.

<u>Rigid 3D registration for 3D image stacks acquired at different time points</u>

To find the largest similarity (e.g., intensity localization and patterns) between two 3D stacks, representative feature points were extracted from the source and target 3D image stacks in 3D space. To achieve this, we firstly extracted 3D point clouds of nerve fibers from converted 8-bit high-resolution 3D image stacks. We then estimated the 3D registration parameters from the extracted 3D point clouds based on the iterative closest point algorithm described below. According to the ICP algorithm, the rigid pairwise local surface registration algorithm can be described as follows. The 3D registration method seeks to find the best transformation T that relates two entities P and Q whose 3D point clouds are given by R_P and R_Q, respectively. The goal is to find T_P such that the objective function $J(R_P, R_Q)$ is minimized,

$$J(R_P, R_Q) = \sum_{P \in R_P} \|\mathrm{T}_P - \Psi(\mathrm{P})\|, \quad (1)$$

where $\Psi: \mathrm{P} \rightarrow \mathrm{Q}$; for $\forall P \in R_P, \Psi(\mathrm{P}) \in R_Q$. The transformation T_P is used to optimally align two point clouds $\mathrm{P} \rightarrow \mathrm{Q}$. In practice, the function $\Psi(\mathrm{P})$ is unknown and needs to compute the Ψ including translation and rotation parameters. The translational part of $\Psi(\mathrm{P})$ brings the center of mass of P to the center of mass of Q. The rotational part of $\Psi(\mathrm{P})$ can be obtained as the unit eigenvector that corresponds to the maximum eigenvalue of a symmetric 4×4 matrix. The solution eigenvector is a unit quaternion description of the rotational part of $\Psi(\mathrm{P})$. During the iterative computation, the positions of the data points are updated by computing the iteration $P_{current} = \Psi(P_{previous})$. While the mean square error is smaller than a given threshold, the iterative optimization converges monotonically to a global minimum. When a good initial value is given, the algorithm can get a global convergence for aligning pairwise 3D point clouds or image 3D stacks.

Non-rigid 3D registration based on Rigid ICP registration

We use the thin-plate-spline (TPS) method for global registration by mapping each local feature point onto its global position. The TPS method has been introduced to 3D geometric deformation processing (Bookstein, 1989, Pottmann, 2006). The TPS has an algebra expressing the dependence of the physical bending energy of a thin plate on point constraints. For interpolating a surface over a fixed set of nodes in plane, the bending energy is a quadratic form in the heights assigned to the surface. On the other

hand, the role of this bending function is similar to the approach of Laplacian-based mesh deformation [6].

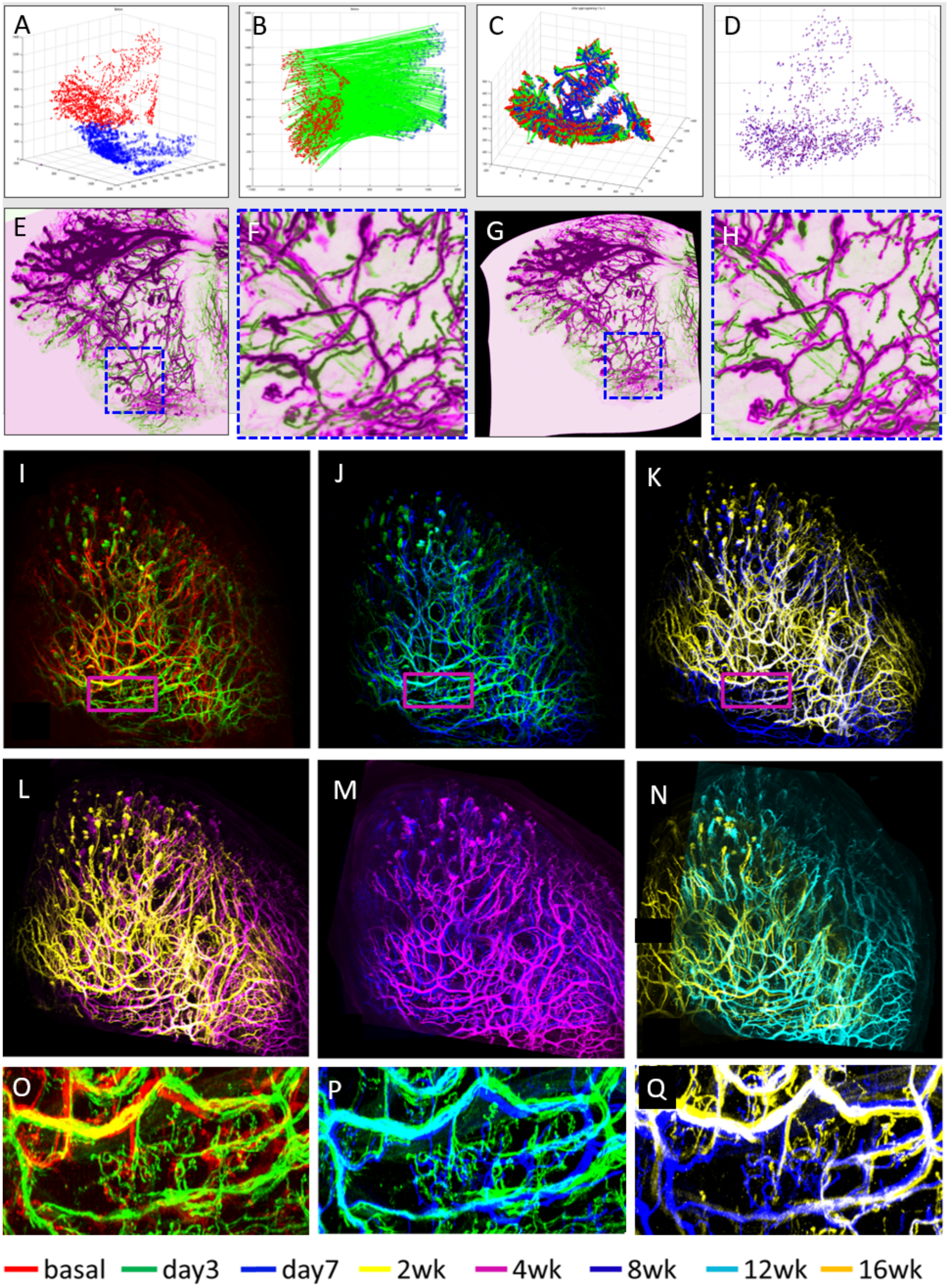

Supplementary Fig. 3 | Stratified 3D registration from rigid alignment to nonrigid alignment in 3D space is based on extracted representative feature points. A, Extracted representative feature points are stored as two vector point clouds from two nerve fibers. **B,**

Estimation of the correspondence between two feature points. **C,** Iterative transformation in 3D space for two corresponding feature point clouds. **D,** Aligned 3D point clouds. **E-H,** The learned 3D registration parameters are directly applied to the original 3D image stack for 3D registration. **I-N,** 3D registration for 3D image stacks acquired from the end phalanges of SNS-GFP mice using our stratified 3D registration approach**. O-Q,** zoom in results from I-K. **Scale bars** (E, G, I-N) 100 μm, (F, H, O-Q) 20μm.

The algorithm of TPS is a non-rigid, globally smooth function including affine and non-affine components. The non-affine warping component means that the sum of squares of all second order partial derivatives is minimized. Such functionality can be used for 3D mapping and it is easily computable. Thus, if $\xi: R^n \rightarrow R$ is an n-dimensional TPS, the bending energy is minimized described in the following,

$$J_\xi = \int (\sum_{i,j} \xi^2_{p_i p_j})\, dp_1 \dots dp_n, \tag{2}$$

In this equation, given two corresponding point sets $P = \{p_1 \cdots p_n\}$ and $Q = \{q_1 \cdots q_n\}$, there is a unique function ξ such that $\xi(p_i) = q_i$ and the bending energy is minimal. Furthermore, this function takes the form $\xi(x) = Ax + BK(x)$ for summing affine component and non-affine warping component.

Where x is a point written in homogeneous coordinates, A is an affine transformation matrix, B is a fixed n-dimensional row vector of non-affine warping parameters constrained to $BP^T = 0$, and $K(x)$ is an n-dimensional column vector. In other words, the TPS kernel contains the information about internal structural relationships in the point-sets and generates a non-rigid warp when combined with the warping coefficient matrix B. Thin-plate splines do not need interpolation. Instead, it can be formulated in a regularization energy function according to (Duchon, 1977),

$$J_\lambda = \frac{1}{n} \sum_{i=1}^{n} |q_i - \xi(p_i)|^2 + \lambda J_\xi. \tag{3}$$

This equation means that the TPS fits a mapping function $\xi(p_i)$ between corresponding point-sets P and Q by minimizing the energy. The first term is a distance measure term between two sets of points. Since $\xi(p_i)$ is not unique, we need smoothness constraints to regulate the mapping. Here, the spline will not be interpolated, but for any fixed λ,

there is a unique minimum. The warping strength depends on the regularization parameter λ. While λ is close to zero, we get an exact alignment of corresponding surface vertices. If λ is zero, interpolation is exact and as it approaches infinity, the resulting TPS surface is reduced to a least squares fitted plane, i.e.,"bending energy"of a plane is 0. For the interpolating case, the thin-plate spline specification provides a linear system of equations.

To apply this equation to solve 3D point sets, based on (Wahba, 1990), we performed a QR factorization to decompose the matrix on Ψ using the approximated equation,

$$AP + B(K + n\lambda I) = G \ , \tag{4}$$

with the condition of $BP^T = 0$. Where K is $n \times n$ and $K_{ij} = G(|p_i - p_j|)$ is a Green's function. We set a small value of λ to improve the stability of numerical computation for this function.

In contrast to previous work, the alignment of numerous local surfaces is globally controlled by the low-resolution global shape model. Since we need to have precise alignments for rigid local scans using the feature prior from the low-resolution global shape model, the spline must be heavily weighted towards interpolation for connecting and aligning slightly-overlapping high-resolution local surface patches. For this reason, we can rely on accurate correspondences to produce a good local surface alignment without global shape distortion or error.

Estimating transformation parameters based on extracted representative feature point clouds

In practice, since one 16-bit image stack consumes 16 GB, an entire end phalanx superstack is around 60 GB. Hence, when aligning superstacks acquired at two time-points, a computer RAM size exceeding 128 GB is required. Furthermore, computing the transformation parameters directly from the superstacks is time-consuming. Therefore, we first perform SIFT or SURF feature detectors in 3D spaces to get representative feature points with only 3D coordinates in 3D spaces. The ICP algorithm can be performed directly using the extracted feature coordinates, e.g. one 3D point cloud is the target point cloud and the other one is a movable point cloud (**Supplementary Fig. 3A-D**). In this way, 3D transformation parameters can be

determined in an efficient way and applied to pairwise superstacks only for pixel-based transformation.

Rigid global 3D alignment and nonrigid local elastic adjustment for optimal 3D registration

In the work diagram, firstly, high-resolution local surfaces are aligned onto the low-resolution global shape model using the rigid ICP method. In this way, the original fidelity of high-resolution local surfaces can be kept after the alignment. Secondly, due to the memory bottleneck problem, we extract representative feature point clouds to reduce the computation load and apply the ICP transformation parameters directly to original image 3D stacks. After the ICP alignment for all pixel-based 3D transformations in the first step, a good starting position for non-rigid TPS alignment in the second step is initialized. In this way, the non-rigid alignment in TPS will be optimized for local adjustment of alignment, shown in **Supplementary Fig. 3E-Q**. Simultaneously, each aligned local nerve fiber and fiber endings are non-rigidly adjusted and refined using a global non-rigid TPS bending algorithm based on initial ICP alignment. 3D alignment from rigid registration to non-rigid 3D registration, for morphology deformation measure and quantitative analysis in 3D and 4D space.

Robustness of 3D registration algorithm

2-photon end phalanx in vivo imaging implies changes between consecutive imaging sessions. In practice, due to the large space of possible 3D nerve fibers changes and deformations, 3D registration of nerve fibers with such partial similarity tends to be challenging, as illustrated in **Supplementary Fig. 4**. Therefore, we had to find and utilize more representative local features and global topological-structure information for 3D registration. Based on the RANSAC algorithm (Fischler et al., 1981), we further optimized the ICP rigid alignment with RANSAC matching and correspondence searching criteria. The alignment can then be obtained by solving a global convex optimization problem with local geometric deformation measure using interactive label information.

In addition, to improve the correspondence searching between two 3D stack pairs, the nerve fibers are decomposed into adaptive patches according to the tree-like structure of the network formed by the nerve fibers. Secondly, the approach is capable of simultaneously computing the mean distribution of fibers in searching 3D space,

represented by a probability density function, from multiple fiber point sets (represented by finite-mixture models), and registering them non-rigidly to this emerging mean distribution shape. Third, the normalized 3D pose and correspondence is served as an initial correspondence for 3D image stack pairs. Since we want to know where and how much of the changes happen, we need to align these 3D datasets using existing partial similarity features including global topology information and local feature similarity information with Z-depth information (**Supplementary Fig. 4**).

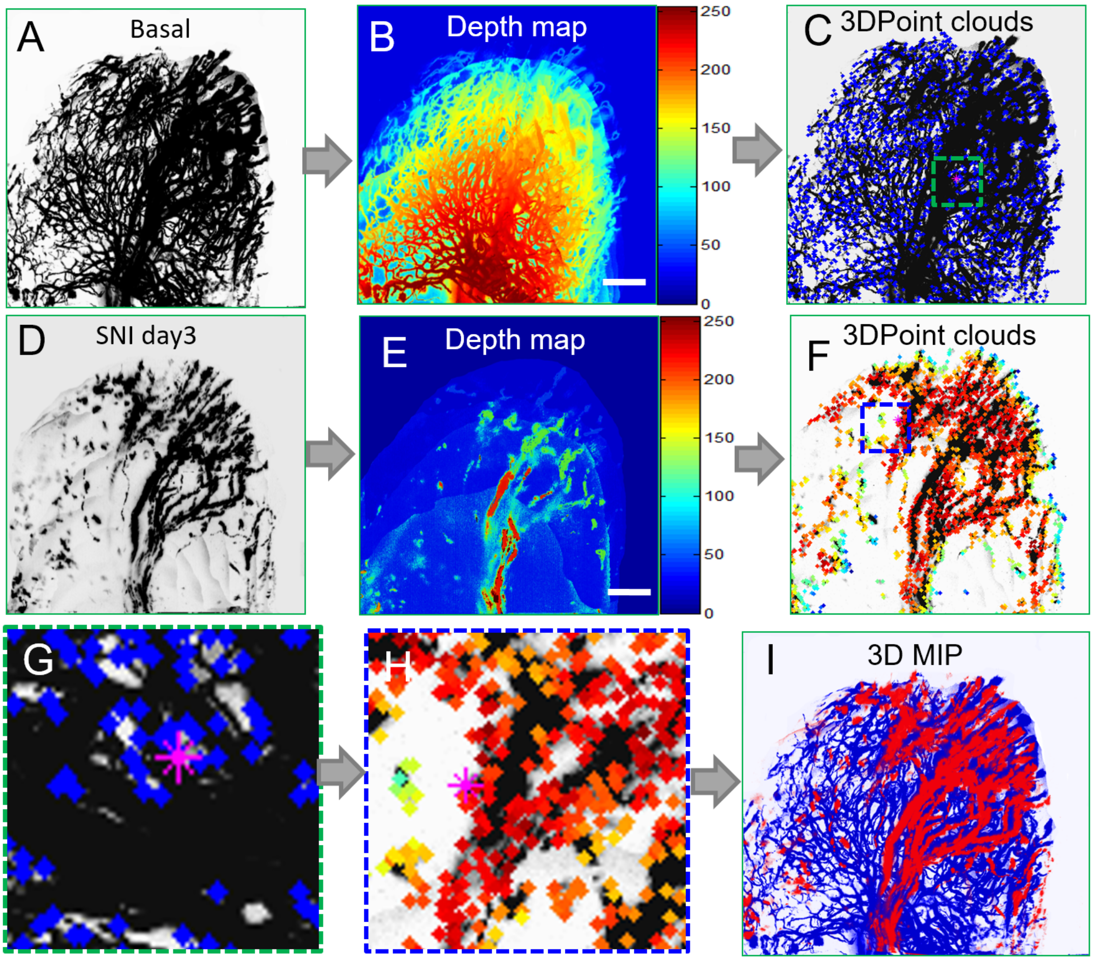

Supplementary Fig. 4. Partial similarity 3D registration using depth-encoding map for generating 3D point clouds with sparse label information. A and D, MIPs from Thy1-YFP mice from basal and SNI day3, **B and E**, Depth-encoding map following the scanning depth from image 3D stacks for generating point clouds with Z-coordinates. **C and F**, 3D point clouds shown in 2D with label information. **G and H,** magnified ROIs shown in C and D, label points shown in magenta. **I,** 3D aligned data based on our method shown in 2D MIP. Scale bar, 100 μm.

The approach is also capable of handling large changes of nerve fibers, in which each test data is represented as compact training features. In addition, it is faster than state-of-the-art approaches and achieves high-quality registrations of two neural fibers with growing changes and with different acquisition angles.

Estimation of maximum common volume (MCV) for image 3D stacks acquired at multiple time-steps in vivo

Shifts in the imaging volume can occur with every imaging session, posing the challenge to crop all superstacks to the same common overlapping volume in order to prevent biases in subsequent quantitative analyses. To solve this problem, we combined two registration workflows to find the best and maximum common regions in each superstack of a time series. We combined the all-to-one 3D registration and pairwise 3D registration to find the MCV (**Supplementary Fig. 5A**). Subsequently, changes in nerve fibers were certain to arise from the same MCV, so changes could not arise artificially from non-overlapping volumes.

V. Automated neuronal segmentation, tracing and statistical neural network analysis

Automated and accurate neural fiber tracing and analysis

Many regularization methods work by pruning noisy branches of the medial axis (Li et al. 2015; Yan et al. 2016), which requires an initial approximation of the medial axis. Based on our datasets and previous studies (Amenta, 2001, Andrea et al, 2016, Huang et al, 2016, Siddiqi and Pizer 2008; Tagliasacchi et al. 2016), we developed a data-driven and task-oriented nerve fiber 3D tracing approach.

Our method rests on two insights on neuronal fiber tracing. Firstly, we show that the medial axis representing shape and structure of a neural fiber can be well approximated, both topologically and geometrically. Second, we show that our approach can provide not only a topologically correct and geometrically convergent approximation from global-to-local optimization, but also magnifies the detection sensitivity of weak nerve fiber signals in deep scanning layers. It is also one of the main differences between our approach and state-of-the-arts approaches in that we can largely trace and analyze discontinuous or broken fibers in the end phalanx (**Supplementary Fig. 5B-G**).

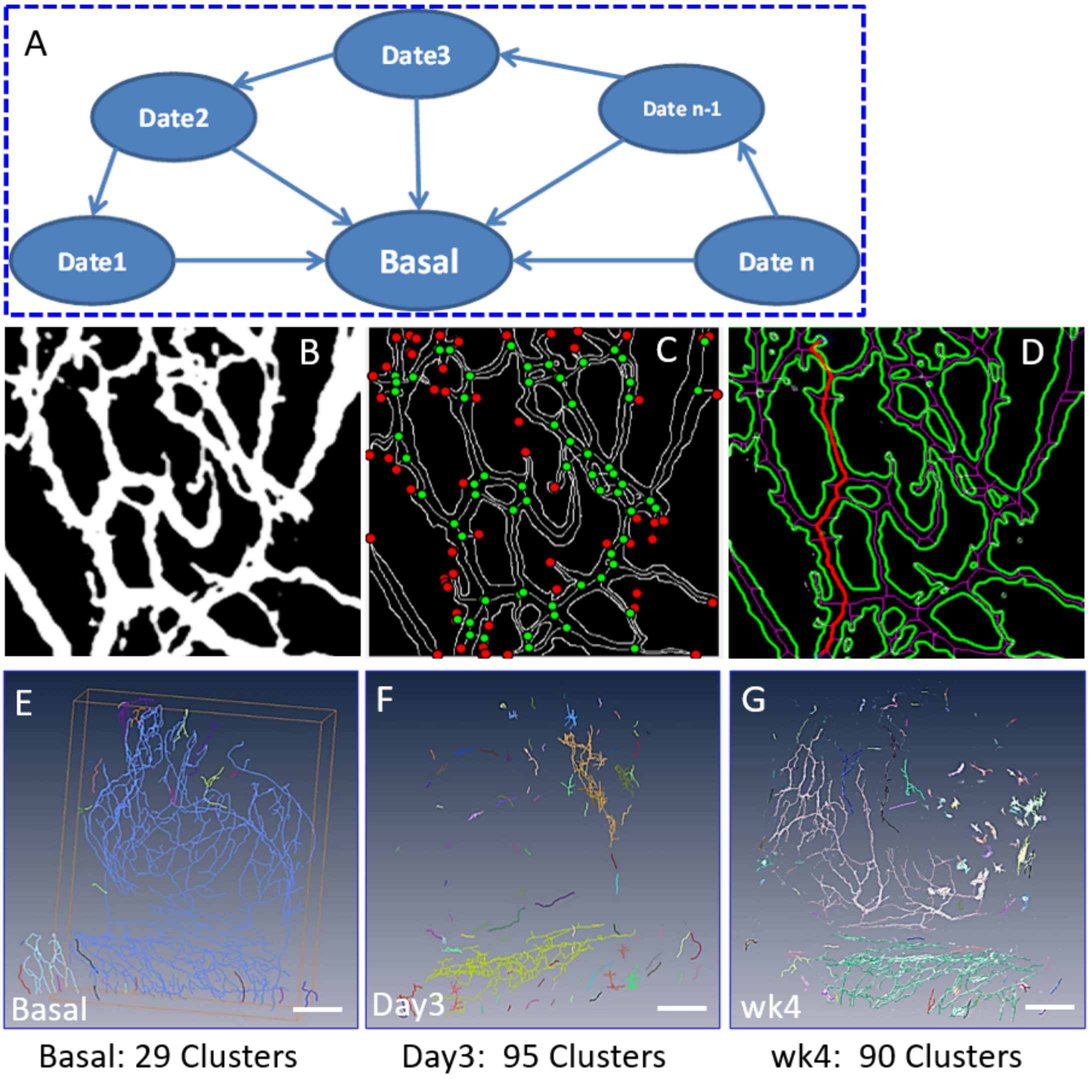

Supplementary Fig. 5 |Tracing, geodesic quantitative measure and clustering of neural fibers in an end phalanx. A, All-to-one 3D registration and pairwise 3D registration and cropping of the maximum common volume of each 3D image stack at each time-step. **B,** Binary representation of nerve fibers. **C,** Tracing of nerve fibers, red dots represent endings and green dots show junctions and branches. **D,** The shortest geodesic distance measures fiber path quantitatively (red line). **E - G,** 3D skeletons of nerve fibers at basal, day3 and week4. Fiber groups that are not connected or are reconnected during the regeneration phase (represented in different colors). Scale bars (D, E, F), 100 μm.

<u>Medial axis and Hausdorff distance</u>

Medial axis. Consider a bounded open set Ω of R^n. The medial axis M is the set of points of Ω that are closest to two or more points on the boundary of Ω, or $\partial\Omega$. More precisely, for any $x \in \Omega$, let $\Gamma(x)$ be the set of its closest points on the boundary,

$$\Gamma(x) = \{y \in \partial\Omega | d(x,y) = d(x,\partial\Omega), \quad (1)$$

where d is the Euclidean distance. The medial axis is defined as $M = \{x \in O \mid |\Gamma(x)| \geq 2\}$. The medial axis of an n-dimensional set is generally a $(n-1)$ −dimensional structure. For $n = 3$, M is made up of 2-dimensional manifolds glued at non-manifold curves (called seams) and points (called junctions). Since each point of M is equidistant to at least two locations on the boundary, M is centered within the fiber shape and captures local geometrical characteristics, e.g., symmetries and diameters. The medial axis has also been shown to be a homotopical equivalent to the open set Ω (Lieutier etal., 2003), which means that the two structures have the same set of topological features, such as holes, tunnels, and connected components.

Hausdorff distance. To measure the distance between two sets V_1, V_2, we use the symmetric Hausdorff distance $d_H$defined as,

$$d_H(V_1, V_2) = \max(\sup_{x \in A} d(x, V_2), \sup_{x \in B} d(x, V_1)). \quad (2)$$

Chazal (Chazal et al., 2005) show that, even though the medial axis is highly sensitive to boundary perturbations, a subset of the medial axis enjoys certain stability properties when the perturbation is bounded by the Hausdorff distance.

Incremental and ensemble L_1-regualrization for accurate 3D nerve fiber tracing

We extended L_1-regularization for computing 3D skeletons of nerve fibers and also magnifying the tracing capability for weak fibers in the deep layers. Given an unorganized and unoriented set of points $V = \{v_j\} \subset R^3$, $j \in J$, we construct the following definition for incremental and ensemble computing the optimal 3D medial skeletons that leads to an optimal set of projection points $X = \{x_i\}, i \in I$,

Enhancement of medial axis detection in 3D spaces using ensemble tracing prior

$$argmin \sum_{i \in I} \sum_{j \in J} \|x_i - v_j\| \xi(\|x_i - v_j\|) + R(X), \quad (3)$$

Where the first term is a localized median points of V, the second term $R(X)$ regularizes the local multiple point distribution of X, I indexes the set of projected points X, and J indexes the set of input points V. The weight function $\xi(r) = e^{-r^2/(h/2)^2}$ is a smooth function with support radius h defining the size of the supporting local neighborhood

for incrementally increasing the single tracing median skeleton in a L_1-regularization iterative optimization way.

The main advantage of definition (3) is that it does not place strong requirements on the quality of the set of points representing the input fiber points. In particular, it can be directly applied to raw multiple tracing point clouds with a scanning depth of 700 layers (e.g., 700 μm) and our approach leads to magnifying the detection sensitivity of neural fibers in a 3D skeleton.

Applying the equation (3) tends to yield a sparse distribution, where the local centers are accumulated into a set of point clusters. To avoid such clustering and maintain the proper medial geometry representation, further accumulation needs to be prevented once points are already contracted onto their local center positions. This is achieved using $R(X)$ in (1), which adds a repulsion force whenever a skeleton branch is formed locally.

For detecting and refining the branches of neural fibers in skeleton computing, we adopted classical weighted PCA to detect the formation of skeleton branches and extended the method of mean shift (Fukunaga and Hostetler 1975, Grillenzoni, 2018) into 3D space. Mean shift is an iterative procedure that shifts each data point to the weighted average of data points in its neighborhood.

Iterative reconstruction of the broken skeleton in broken fibers with weak signals is specially developed for our datasets. We first use neighborhood data points to estimate a representative central median skeleton in a weighted mean shift adjustment in our regularization function. We also apply a simple 3D buffer to guide the ensemble extension of the median skeleton in broken region or volume as illustrated in **Supplementary Fig. 6**. Inside the blue buffer volume in **Supplementary Fig. 6 H-L**, the ensemble growing and mean-shift will connect the broken skeleton by using weak fiber signals. These weak fiber signals will give strong weight inside the buffer volume for connecting the broken in a locally-weighted ensemble construction way. Following the buffer volume, the computation will incrementally connect the broken fibers with weak fiber signals in a shift window to the global volume.

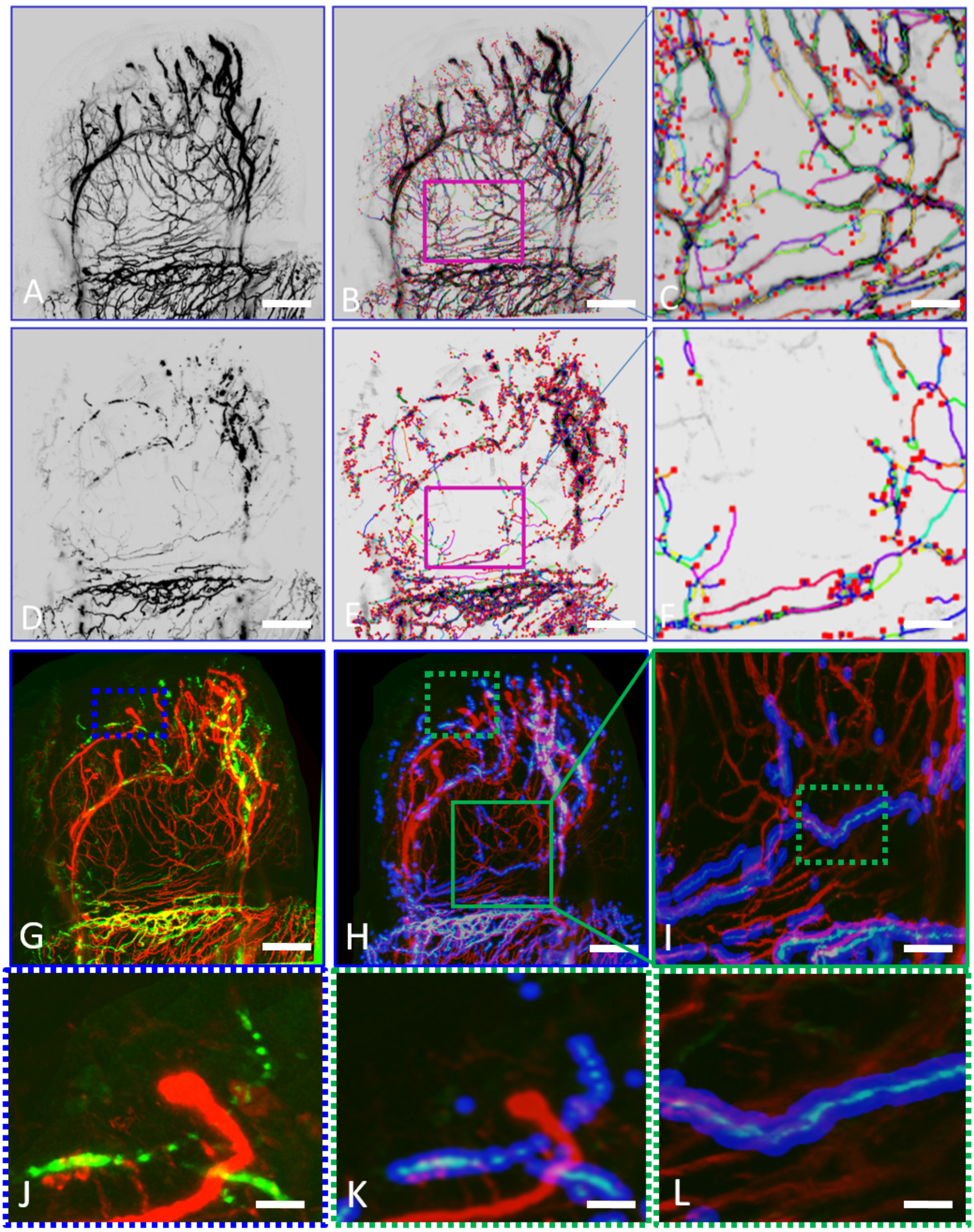

Supplementary Fig. 6 | 3D tracing and quantitative measure of normal nerve fibers and broken nerve fibers. **A,** Nerve fiber network in the basal state. **B,** Fiber skeletons (different colors) and terminating structures (red dots). **C,** magnified view of ROI shown in B. **D,** Fiber network three days after SNI surgery. **E and F,** Traced skeletons. **G,** after 3D registration of basal and day3 nerve fibers. **H,** Construction of a buffer volume of day3 nerve fibers for connecting and tracing broken fibers within a given buffer threshold. **I-L**, Magnfications of images shown in G and H. The buffer volumes (blue) serve as constraints for connecting and

tracing the skeleton of broken fibers. Scale bars (A, B, D, E, G, H), 100 μm. Scale bars (C, F, I), 25 μm, 20 μm in (J, K, L).

As a result, our algorithm may produce skeletons with certain global and local constraints with high-fidelity and correct topology as highlighted in **Supplementary Fig. 5E-G.** We identify the number of traced fiber clusters during the growth period. The geodesic distance measure can largely help to measure the broken distance in a broken volume which is often by the constraints of utilizing the buffer volume. In this way, we can also add more information for optimal reconstruction of the broken fibers, e.g., normal of fiber surface patches in **Supplementary Fig. 6,** and outward directions directly derived from spatial-temporal fiber changes.

<u>Construction of 3D graph for spatially-localized and topological network analysis</u>

To achieve cluster analyses of 3D fiber networks, localization of fiber changes, as well as activity-dependent structural plasticity analysis were developed. These algorithms include:

- Analysis of connectivity and fibers using graph $G=(V,E)$, e.g., dendrogram, and make the graph more bio-informative,
- Accurate 3D registration in a sliding 3D window to measure fine structures and branching changes in 4D space.
- Localize and measure dynamic changes of fine structure of dendritic filopodia and small branches in 4D space.
- Dynamic change of neural pattern and neural connections (clusters, undirected graph, data-driven and task-oriented quantitative measure).

A graph network-based cluster analysis in 3D space revealed the location and quantity of broken fiber networks, location and quantity of regenerated fiber networks, etc. Furthermore, we construct the undirected graph network to represent the neural fibers in a graph network. In this structural graph network, we can measure and localize the changes not only using Euclidean distance measure (e.g., 3D registration using Euclidean distance) but also using geodesic distance measure of fiber length, fiber connectivity and localize the fine structural changes in a converted graph network.

Construction of 3D graph network

In this step, the traced 3D skeleton was converted into a graph network to measure neural fiber plasticity and changes in 4D space. The network topology is represented as a sparse connectivity matrix A, where $a_{i,j}$ is the length of the shortest link between nodes v_i and v_j, or 0 if no such link exists. The size of the matrix corresponds to the number of nodes N and defines the graph size. The edge density, also called connectivity, is calculated as the number of existing edges E divided by the number of possible edges in an undirected graph, $N(N-1)/2$. The Euler characteristic for graphs is defined as the number of edges minus the number of nodes, $N-E$ (Mecke and Stoyan 2001). The degree, or weight, of a node is the number of edges connected to it.

Global connectivity and local connectivity measured in graph network using geodesic measure.

Here we use the geodesic path as the shortest path and distance along the fibers. The shortest path (SP) and the average shortest path (ASP), which measure the typical separation between two nodes in the network, is derived by taking the mean of all shortest paths between any two nodes. The topological architecture of the network allows quantifying the efficiency of the network for information transfer based mainly on geodesic measures. We found that the shortest paths between nodes in the network run along the fibers with most high-centrality nodes clustered together. The centrality of a connected node is the fraction of all such shortest paths in the network running through that node. This analysis provides insights into neural plasticity changes using local to global principles into a large, interconnected network during neural degeneration and regeneration.

3D volume computing and surface reconstruction of neural fibers

Different types of quantitative measures such as volume, tracing and segmentation can be applied not only for neural geometrical and topological analysis, but also for understanding structure and function of neural plasticity. Here we first segment and measure the volume of neural fibers in 3D spaces after removing epidermal autofluorescence and appendages. Nerve fibers were reconstructed and measured based on surface reconstruction using number of patches. Different types of neural fibers in the end phalanx can also be segregated and segmented based on the combination of

fiber morphological thickness and connectivity properties. For example, thick fibers are segregated from thin fibers in the end phalanx of Thy1-YFP mice.

VI. Quantitative analysis of structural plasticity in SNS-mGFP and Thy1-YFP mice

Differences of nerve fiber morphology between SNS-mGFP and Thy1-YFP mouse lines are illustrated in **Fig. 1**. In the 3D spatial domain, these differences mainly include the distribution density of fibers, length, thickness of fibers and the location of fibers. Therefore, the segmentation and tracing algorithms were adjusted accordingly as detailed in the following.

<u>4D analysis of epidermal fibers in SNS-mGFP datasets</u>

The analysis workflow designed to capture changes of thin epidermal fibers is described in the following (**Supplementary Fig. 7**). Epidermal fibers and deep fibers in SNS-mGFP mice paw are classified so that changes of epidermal fibers can be quantitatively measured and localized. The workflow applied to a complete SNS-mGFP dataset acquired at all different timesteps.

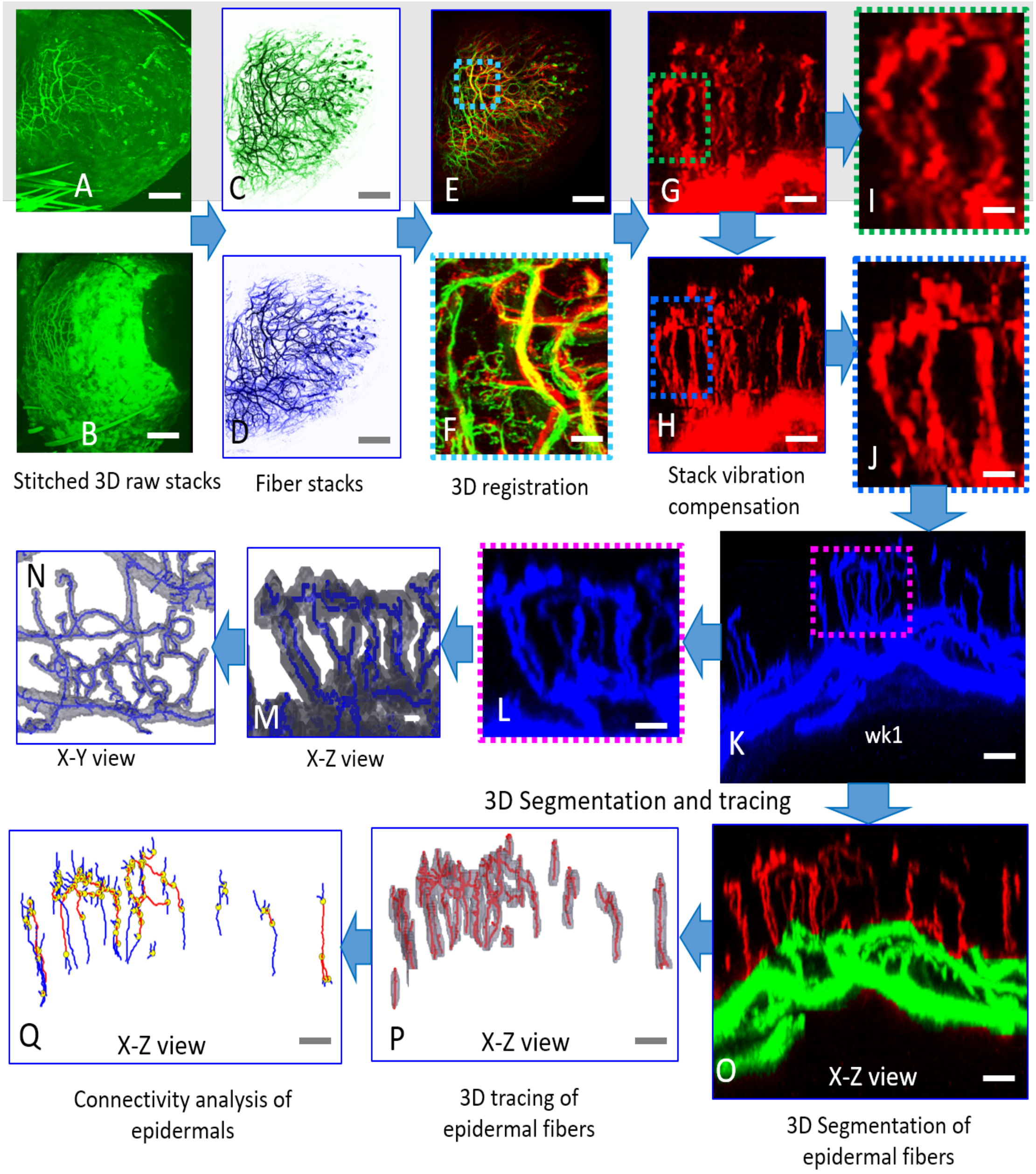

Supplementary Fig. 7 | Quantitative analysis of epidermal fibers in SNS-mGFP mice. A and B, Two 3D raw image stacks acquired at different time points. **C and D,** Corresponding MIPs after applying steps I to III of the overall workflow. **E,** 3D aligned image stacks. **F,** magnified view of blue box in E. **G,** XZ-view of epidermal and deep nerve fibers with distortions generated by heat-beat vibration. **H,** Z-direction alignment again for reducing vibration of heart beat in mice. **I and J,** Zoom in ROI from G and H. **K,** XZ-view for epidermal and deep fibers acquired at the time-step wk1. **L,** X-Z view of ROI volume from K. **M-N,** 3D skeleton of epidermal and deep fibers. **O,** Segmentation of epidermal (red) and deep fibers (green). **P,** Quantitative measure of epidermal fibers including length of and volume of

epidermal fibers. **Q,** Graph networks of epidermal fibers for statistical analysis. Scale bars (A-E), 100 μm. Scale bars (F, G, H, K, O, P, Q), 35 μm.

References

Arici, T. and S. Dikbas, and Y. Altunbasak. A histogram modification framework and its application for image contrast enhancement. IEEE Transactions on Image Processing, 18(9):1921–1935, 2009

Amenta, N., and Kolluri, R. K. The medial axis of a union of balls. Comput. Geom. 20, 1-2, 25-37,2001.

Bay, Herbert and Tinne Tuytelaars and Luc Van Gool, SURF: Speeded Up Robust Features (Memento vom 19. März 2015 im Internet Archive) (PDF; 723 kB), Proceedings of the 9th European Conference on Computer Vision, Springer Verlag, 2006

Bergevin, R. and M. Soucy, H. Gagnon, and D. Laurendeau. Towards a general multi-view registration technique. IEEE Trans. on PAMI, 18(5):540–547, 1996.

Besl, P. and N. McKay. A method for registration of 3D shapes. IEEE Trans. on PAMI, 14(2):239-256, 1992.

Bookstein. F. L., Principal warps: Thin-plate splines and the decomposition of deformations. IEEE Trans. on PAMI, 11(6): 567-585, 1989.

Boykov, Y. and Veksler, O. , R. Zabih, Fast approximate energy minimization via graph cuts, Proc. IEEE Trans. Pattern Anal. Mach. Intell. 23 (11) (2001) 1222-1239.

Burt, P. and Adelson, E. The Laplacian pyramid as a compact image code. IEEE Transactions on Communications, 31(4):532-540, 1983.

Chennubhotla, C. and A. Jepson, "Hierarchical Eigensolver for Transition Matrices in Spectral Methods," Advances in Neural Information Processing Systems 17, L.K. Saul, Y. Weiss, and L. Bottou, eds. MIT Press, pp. 273-280, 2005.

Choi, H., Lee, C. No-reference image quality metric based on image classification. EURASIP J. Adv. Signal Process. 2011, 65 (2011). https://doi.org/10.1186/1687-6180-2011-65

Duchon. J., Splines minimizing rotation-invariant seminorms in sobolev spaces. In Constructive Theory of Functions of Several Variables, pages 85–100. Springer-Verlag, 1977.

Fan M, Zhang X, Du L, Chen L, Tao D. Semi-Supervised Learning Through Label Propagation on Geodesics. IEEE Trans Cybern. 2018 May;48(5):1486-1499.

Fukunaga, K., The Estiamtion of the Gradient of a Density Function with Applications in Pattern Recognition in, *IEEE Transactions on Informaiton Theory*, vol. A, no. 2, pp. 32-40, 1975.

Grady, L. and T. Schiwietz, S. Aharon, and R. Westermann, “Random Walks for Interactive Alpha-Matting,” Proc. Fifth IASTED Int’l Conf. Visualization, Imaging, and Image Processing, 2005.

Grillenzoni, C., Sequential mean shift algorithms for space–time point data. Environ Earth Sci 77, 336 (2018).

Gracias, N. and Mahoor, M. and S. Negahdaripour and A. Gleason , Fast image blending using watersheds and graph cuts, Image and Vision Computing 27(5):597-607, 2009

Huang, H. and Shihao Wu, Daniel Cohen-Or, Minglun Gong, Hao Zhang, Guiqing Li, and Baoquan Chen, "L1-Medial Skeleton of Point Cloud", ACM Trans. on Graphics, 2013

Kolmogorov, V. and R. Zabih, What energy functions can be minimized via graph cuts? IEEE Trans Pattern Anal. Mach. Intell. 26 (2) (2004) 147-159.

Kuner, Rohini., Central mechanisms of pathological pain, Nature Medicine,16,1258-1266, 2010)

Lee T C, and Kashyap R L and Chu C N, Building skeleton models via 3D medial surface axis thinning algorithms, CVGIP, Graph. Models Image Process, 1994

Levin, Anat and Dani Lischinski, and Yair Weiss, A Closed-Form Solution to Natural Image Matting, IEEE Transactions on Pattern Analysis and Machine Intelligence, vol. 30, no. 2, Feb 2008

Lowe, David G., Distinctive Image Features from Scale-Invariant Key points, International Journal of Computer Vision. Band 60, Nr. 2, Seiten 91-110, 2004

Martin A. Fischler & Robert C. Bolles (June 1981). "Random Sample Consensus: A Paradigm for Model Fitting with Applications to Image Analysis and Automated Cartography" (PDF). Comm. ACM. 24 (6): 381-395.

Mecke, K. R. and Stoyan, D., Statistical Physics and Spatial Statistics-The Art of Analyzing and Modeling Spatial Structures, Lecture Notes in Physics, Vol. 554 (Springer, 2000).

Mittal, A., A. K. Moorthy, and A. C. Bovik. "No-Reference Image Quality Assessment in the Spatial Domain." *IEEE Transactions on Image Processing*. Vol. 21, Number 12, December 2012, pp. 4695-4708.

Mittal, A., R. Soundararajan, and A. C. Bovik. "Making a Completely Blind Image Quality Analyzer." *IEEE Signal Processing Letters*. Vol. 22, Number 3, March 2013, pp. 209-212.

Pizer, S. M. and E. P. Amburn, J. D. Austin, R. Cromartie, A. Geselowitz, T. Greer, B. ter Haar Romeny, J. B. Zimmerman, and K. Zuiderveld. Adaptive histogram equalization and its variations. Computer Vision, Graphics, and Image Processing, 39(3):355-368, 1987

Pottmann, H., and Q.-X. HuangG, Y.-L. Yang, and S.-M. Hu. Geometry and convergence analysis of algorithms for registration of 3D shapes. IJCV, 67(3):277-296, 2006.

Szeliski, R. and S. Lavallee. Matching 3-D anatomical surfaces with non-rigid deformations using octree-splines. IJCV, 18(2):171-186, 1996.

Tagliasacchi, A. and Delame, Thomas, Michela Spagnuolo, Nina Amenta, Alexandru Telea, 3D Skeletons: A State-of-the-Art Report , EUROGRAPHICS, 2016

Venkatanath, N. and D. Praneeth, Bh. M. Chandrasekhar, S. and S. S. Medasani., Blind Image Quality Evaluation Using Perception Based Features, In *Proceedings of the 21st National Conference on Communications* (NCC). Piscataway, NJ: IEEE, 2015.

Wahba. G., Spline Models for Observational Data, volume 59. SIAM, 1990.

Wang G, Zuluaga MA, Li W, et al. DeepIGeoS: A Deep Interactive Geodesic Framework for Medical Image Segmentation. IEEE Trans Pattern Anal Mach Intell. 2019;41(7):1559-1572. doi:10.1109/TPAMI.2018.2840695

Zheng, H., Saupe, D., Complex 3D shape recovery using hybrid geometric shape features in a hierarchical shape segmentation approach, IEEE 3D Digital Imaging and Modeling (3DIM 2009), pp. 1662-1669, 2009.

Zhu, Xiaojin, and Zoubin Ghahramani. "Learning from Labeled and Unlabeled Data with Label Propagation." CMU CALD tech report CMU-CALD-02-107. 2002.

Supplementary note 3: Tyrosine hydroxylase (TH)-expressing fibers

TH is expressed in all sympathetic and dopaminergic nerve fibers (Brumovsky, Pain 2016). Accordingly, anti-TH immunohistochemistry in glabrous hindpaw plantar skin of sham-treated SNS-mGFP revealed their localization in deeper parts of the dermis, consistent with adrenergic innervation of sweat glands. A sub-population of mGFP-positive sensory fibers were also immunoreactive for TH in the dermis. This is consistent with the current literature which indicates that TH-expressing neurons in the DRG are C-LTMRs, which is a subset of non-peptidergic neuronal population expressing Ret and GINIP, TafA4 and Vglut3, but distinct from the IB4-binding or MGPRD population (Brumovsky, Pain 2016; Delfini et al. 2013; Piers et al. 2015; Usoskin et al. 2015). TH-positive C-LTMRs and are known to robustly innervate hair follicles in hairy skin with very little innervation of glabrous skin reported (Brumovsky, Pain 2016). Accordingly, none of the 8 sham-treated SNS-mGFP mice tested show TH-immunolabelling at the dermal-epidermal border or in the epidermis of the glabrous skin (Extended Data Fig. 9a). A similar pattern of TH-expression was observed in the denervated tibial territory of 6 out of 8 SNS-mGFP mice tested at 42 weeks post-SNI (Extended Data Fig. 9c). In contrast, the remaining two mice showed presence of anti-TH immunolabelling, which was partially overlapping with mGFP expression, at the dermis-epidermis border and partly invading into the epidermis (Extended Data Fig.

9b). This raises the possibility that C-LTMRs may also collaterally sprout from hairy skin into denervated glabrous skin. Given that they are a sub-population of the SNS-fibers (Nav1.8-expressing) that were ablated in the functional experiments shown in Fig. 4, it cannot be discounted that they contribute to mechanical allodynia in the tibial denervated territory. However, this is not likely given the low incidence of the putative collateral sprouting of TH fibers observed as well as the lack of functional indications thereof in the skin-nerve recordings we performed. In the absence of hair shafts, which they normally act as a lever for transmitting force to the nerve ending, it is unclear whether C-LTMRs sprouting into the epidermis as free nerve endings would show their typical low mechanical thresholds for activation. Moreover, TH-expressing DRG neurons have been recently suggested to also include non-C-LTMR neurons (Morelli et al. BioRxiv; doi: https://doi.org/10.1101/2020.01.17.909960). At present, these findings therefore do not present a strong case for a functional contribution of C-LTMRs to the late-onset mechanical hypersensitivity developing in the denervated tibial territory post-SNI.

References:

Brumovsky PR. Dorsal root ganglion neurons and tyrosine hydroxylase--an intriguing association with implications for sensation and pain. Pain. 2016 Feb;157(2):314-20.

Delfini MC, Mantilleri A, Gaillard S, Hao J, Reynders A, Malapert P, Alonso S, François A, Barrere C, Seal R, Landry M, Eschallier A, Alloui A, Bourinet E, Delmas P, Le Feuvre Y, Moqrich A. TAFA4, a chemokine-like protein, modulates injury-induced mechanical and chemical pain hypersensitivity in mice. Cell Rep. 2013 Oct 31;5(2):378-88.

Morelli C, Castaldi L , Brown SJ, Streich LL, Websdale A, Taberner FJ, Cerreti B, Barenghi A, Blum KM, Sawitzke J, Frank T, Steffens L, Doleschall B, Serrao L, Lechner SG, Prevedel R, Heppenstall PA. Identification of a novel population of peripheral sensory neuron that regulates blood pressure. 2020, doi: https://doi.org/10.1101/2020.01.17.909960

Peirs C, Williams SP, Zhao X, Walsh CE, Gedeon JY, Cagle NE, Goldring AC, Hioki H, Liu Z, Marell PS, Seal RP. Dorsal Horn Circuits for Persistent Mechanical Pain. Neuron. 2015 Aug 19;87(4):797-812.

Usoskin D, Furlan A, Islam S, Abdo H, Lönnerberg P, Lou D, Hjerling-Leffler J, Haeggström J, Kharchenko O, Kharchenko PV, Linnarsson S, Ernfors P. Unbiased classification of

sensory neuron types by large-scale single-cell RNA sequencing. Nat Neurosci. 2015 Jan;18(1):145-53.

Supplementary note 4: Transcriptomics

We performed RNA expression analyses to address whether nociceptors which collaterally sprout from the sural nerve into the denervated tibial territory at 24 weeks post-SNI show molecular differences from the native nociceptors of the tibial nerve which populate and innervate this territory in physiological conditions (sham). The somata of these axons were retrogradely labelled by injection of tracers in a very small volume in the plantar paw skin corresponding to the tibial territory and picked following acute dissociation. During this step, we segregated tracer-labelled neurons as being IB4-positive and IB4-negative depending upon binding with fluorescently labeled-IB4 and subjected them to RNA sequencing as described under methods. As additional controls and comparisons, we also performed similar analyses on somata of fibres that innervate the sural territory in control and neuropathic mice SNI 24 weeks. All raw and annotated gene expression data with corrected p values are uploaded on a public repository European Nucleotide Archive (https://www.ebi.ac.uk/ena) under the accession number PRJEB50184. In the description below, the labels 'tibial' and 'sural' denote the corresponding skin territory. IB4-positive neurons are unequivocally identified as nociceptors in all groups. In case of IB4-negative neurons, although we tried our best to pick small-diameter cells post-dissociation, these can potentially include non-nociceptive neurons, particularly in the control condition for the tibial territory group and both control and SNI conditions in the sural territory group. However, we would like to emphasize that we have also analyzed data in the IB4-negative population and observed that there are no differences which would go against the conclusions derived from the gene expression analyses.

Our main findings are as follows:

1. Although up to 26154 genes were studied in total, the number of regulated genes is very modest (in total 24 in tibial territory and 68 in sural territory), as shown in Extended Fig. 13. This is completely plausible, in our view, since analysis was performed at late stages (6 months post-injury), when local injury and inflammation

pathology is absent and reinnervation has already occurred, so the massive regulation of injury and degradation-related genes that is typical to early stages is not expected. Secondly, previous studies that looked at gene regulation post nerve-injury analyzed all DRG neurons in bulk. This study compared nociceptors in their native state of innervation with nociceptors that had collaterally sprouted and established reinnervation. When comparing gene expression differences, we noticed that the largest differences actually occur between IB4+ and IB4- neurons, which are given by the nature of their cell specialization, in both sham and SNI mice; they are, therefore, addressed separately in the data below. Within the same subpopulation of nociceptors, gene expression did not vary much between the treatment groups.

2. The representation of different sub-classes of nociceptors in the tibial territory post-SNI was compared with sham mice using deconvolution analyses and clustering with marker proteins described by Usoskin et al. (2015). We did not find any significant differences in the relative abundance of the NP1 and NP2 sub-populations and PEP1 and PEP2 sub-populations of peptidergic nociceptors. The PEP3 subclass was represented to a very low extent across the sham samples and not detectable in the SNI samples, but owing to the low abundance in the overall population of nociceptors, this was not significant. A Chi Square analysis of comparison of overall distribution of the 5 sub-populations of nociceptors did not reveal a significant difference (Extended Fig. 13c). This, therefore, argues against dominance of a particular class of nociceptors with altered or special properties amongst the collaterally sprouting nociceptors as a mechanistic basis for mechanical allodynia developing in the denervated sural territory.

3. Modulators of neuronal excitability: As compared to control mice, we did not observe any upregulation of genes encoding reported mediators of peripheral sensitisation in the nociceptors reinnervating the tibial territory in SNI mice (24-26 weeks post-SNI). For example, there was no upregulation of ion-channels transducing sensory stimuli, such as TRP channels, Piezo2 or ATP-gated channels (P2X3), or enhancing membrane excitability, such as diverse sodium channels, calcium channels or potassium channels that have been linked to nociceptor sensitization, amongst others. Data from IB4-positive (putative non-peptidergic nociceptors) and IB4-negative populations are shown below in Table 1 and Table 2, respectively.

Table 1: Expression of key known ion-channels regulating nociceptor excitability, activation and conduction in IB4-positive neurons innervating either the reinnervated tibial territory or sham territory at 24-26 weeks post-SNI or control. None of the differences were statistically significant as per corrected p values (see methods for bioinformatics analyses). CPM: gene counts per million.

	**Sham**	**SNI**	**Sham**	**SNI**
Genes	Tibial (CPM)		Sural (CPM)	
Asic1	2.95±2.7	3.16±1.40	5.11±4.02	0.27±0.27
Asic2	49.94±7.46	42.63±5.72	39.89±8.69	35.98±13.24
Asic3	15.90±10.8	2.89±2.83	58.70±29.44	1.87±1.58
Kcna1	10.71±6.52	12.38±7.79	7.88±4.14	3.96±2.26
Kcna2	42.97±12.27	31.55±11.02	41.23±11.26	17.24±8.88
Kcnc4	27.94±5.24	19.87±4.85	15.85±4.74	42.05±20.37
Kcnk1	22.44±14.91	7.56±2.32	30.50±11.19	4.76±3.07
Kcnk18	20.25±5.80	12.48±3.27	21.49±6.63	31.79±18.41
Kcnk3	4.33±2.66	0.96±0.96	6.52±3.52	7.27±7.13
Kcnk9[#]	0.00±0.00	0.20±0.15	0.00±0.00	0.00±0.00
Kcns2[#]	0.06±0.05	0.33±0.33	0.00±0.00	0.00±0.00
Piezo1	2.80±1.75	2.94±1.21	3.20±1.45	1.37±0.89
Piezo2	138.71±22.20	125.68±14.72	140.49±8.61	125.38±43.74
Scn10a	782.70±22.22	636.19±184.99	754.70±81.76	1131.01±62.34
Scn11a	935.76±49.38	841.05±262.90	869.04±140.67	1345.97±122.89
Scn3a	0.99±0.42	0.09±0.09	1.31±0.66	0.00±0.00
Scn8a	11.46±5.77	20.23±16.59	17.92±9.12	2.22±1.93
Scn9a	158.03±15.21	130.55±31.15	156.14±58.86	178.02±64.87
Trpa1	73.32±15.25	72.98±11.20	48.01±12.95	58.54±22.80
Trpc1	3.29±1.60	4.67±1.78	1.94±0.58	9.21±6.34
Trpc3	116.68±17.41	129.17±24.98	102.77±8.70	152.49±15.19
Trpc4	11.19±5.04	1.66±1.17	6.55±2.63	3.19±1.87
Trpc5	2.58±2.12	2.68±1.00	1.38±0.45	1.11±0.93
Trpc6	32.07±6.32	43.82±7.57	39.53±8.87	49.59±16.11
Trpc7	0.14±0.14	0.00±0.00	2.04±2.04	0.30±0.30
Trpm1	0.04±0.04	0.25±0.25	1.09±1.09	0.00±0.00
Trpm2	4.63±2.51	1.16±0.67	8.93±4.60	3.58±3.58
Trpm3	21.53±1.75	26.18±3.61	18.68±4.66	24.09±5.69
Trpm4	36.32±10.11	17.66±2.77	28.97±7.47	35.19±8.62
Trpm5	1.82±0.45	1.16±0.50	0.75±0.35	0.67±0.23
Trpm6	2.95±2.07	0.25±0.25	0.17±0.17	0.30±0.30
Trpm7	20.27±6.27	15.94±5.10	13.16±2.08	16.26±5.66
Trpm8[#]	0.00±0.00	0.03±0.03	0.91±0.91	0.04±0.04
Trpv1	56.32±24.96	33.70±13.89	93.00±41.58	42.31±14.23
Trpv2	128.62±23.84	156.20±13.96	163.83±48.89	112.89±35.35
Trpv3[#]	0.35±0.20	0.00±0.00	0.19±0.19	0.13±0.13

Trpv4#	0.00±0.00	0.13±0.13	0.00±0.00	0.11±0.11
Trpv6#	0.21±0.21	0.00±0.00	0.00±0.00	0.22±0.22

# refers to genes with very low and unreliable counts.

Table 2: Expression of key known ion-channels regulating nociceptor excitability, activation and conduction in IB4-negative neurons innervating either the reinnervated tibial territory or sham territory at 24-26 weeks post-SNI or control. None of the differences were statistically significant as per corrected p values (see methods for bioinformatics analyses). CPM: gene counts per million.

	**Sham**	**SNI**	**Sham**	**SNI**
Genes	Tibial (CPM)		Sural (CPM)	
Asic1	30.21±5.79	29.69±7.12	24.75±7.26	32.14±4.16
Asic2	28.10±7.61	27.94±4.34	63.07±11.77	88.10±10.49
Asic3	143.79±60.80	182.28±40.50	193.89±27.15	222.81±40.58
Kcna1	70.61±27.66	80.08±28.51	90.47±19.50	52.35±7.73
Kcna2	138.91±16.76	175.64±45.23	148.93±18.16	108.29±5.10
Kcnc4	42.97±16.07	38.51±6.94	17.10±6.79	21.92±3.70
Kcnd2	1.22±1.22	0.50±0.29	1.47±1.39	1.01±0.24
Kcnk1	84.06±22.45	153.09±19.43	73.09±25.41	144.98±19.44
Kcnk18	15.42±5.45	20.63±6.85	18.64±2.72	18.12±3.36
Kcnk3	13.21±4.65	10.74±2.78	2.57±1.53	4.38±1.20
Kcns2	1.05±0.93	0.41±0.31	0.04±0.04	0.00±0.00
Piezo1	1.91±1.23	1.89±1.34	0.13±0.08	1.81±0.58
Piezo2	211.22±28.85	214.19±18.63	232.04±48.52	116.20±23.86
Scn10a	351.17±109.71	317.17±73.88	369.18±82.57	310.24±40.76
Scn11a	199.98±101.29	85.04±21.11	189.45±36.37	139.48±30.44
Scn3a	3.70±1.72	3.13±2.03	6.15±3.20	2.30±0.44
Scn8a	101.06±19.59	122.96±29.99	103.48±7.80	59.88±3.93
Scn9a	153.86±28.85	147.21±12.02	153.28±27.91	103.42±4.55
Trpa1	20.79±16.47	17.27±7.25	7.19±3.14	19.82±7.15
Trpc1	5.61±1.36	5.25±1.61	10.40±2.43	4.99±0.39
Trpc3	28.30±20.68	4.00±0.85	6.54±3.00	11.89±4.10
Trpc4	1.29±1.29	1.68±0.79	0.00±0.00	0.37±0.22
Trpc5	1.45±1.45	0.41±0.41	0.14±0.14	1.77±0.49
Trpc6	1.51±0.82	1.95±1.50	0.65±0.50	2.89±1.19
Trpc7	2.84±1.81	6.63±2.68	1.27±0.74	0.95±0.48
Trpm1#	0.00±0.00	0.87±0.56	0.14±0.10	0.14±0.14
Trpm2	19.37±4.23	14.43±2.56	14.07±1.95	17.68±2.34
Trpm3	15.43±7.01	13.59±4.98	8.62±3.70	7.15±1.77
Trpm4	12.89±4.68	12.37±3.31	17.76±3.69	14.95±3.21
Trpm5	0.73±0.31	1.46±0.66	1.88±0.44	1.42±0.36
Trpm6	0.00±0.00	1.43±0.52	2.22±0.93	0.61±0.45

Trpm7	18.05±2.57	19.36±3.37	17.90±1.57	19.74±1.53
Trpm8	0.36±0.36	1.89±0.67	3.06±2.42	0.70±0.70
Trpv1	69.68±27.37	85.74±15.68	34.28±18.39	50.84±12.42
Trpv2	66.83±22.13	87.07±23.06	59.47±11.51	73.78±7.56
Trpv3	0.10±0.10	0.54±0.39	37.71±9.05	21.32±1.47
Trpv4	0.80±0.80	0.00±0.00	1.64±1.64	0.54±0.35
Trpv6[#]	0.00±0.00	0.63±0.54	0.00±0.00	0.06±0.06

[#] refers to genes with very low and unreliable counts.

Moreover, in DRG neuronal somata with terminations in sural territory, we also did not find major indications of gene expression changes indicative of classical nociceptor sensitization at this chronic time point post-SNI. Genes encoding the sodium channel Nav1.1 (*Scn1a*) and the key growth factor receptors, TrkA (*Nrtk1*) and TrkB (*Ntrk2*), were actually significantly downregulated (rather than being upregulated) in sural territory neurons of SNI mice as compared to sham mice. We conclude therefore that nociceptor sensitization phenomena induced by molecular mediators that are hallmarks of other types of pain, such as acute and chronic inflammatory pain, are not causing the observed change in mechanical thresholds upon reinnervation.

4. In DRG neuronal somata of nociceptors that collaterally sprouted into the tibial territory at 24-26 weeks post-SNI, instead of finding regulation of genes linked to peripheral sensitization as explained above, we found that most of the genes that were significantly differentially expressed in collaterally sprouting nociceptors over sham conditions are directly linked to structural changes, such as neurite outgrowth, axonal pathfinding, neuroprotection and axonal survival, or belong to families that have been ascribed these functions. Some of these genes are indeed downstream of NGF and reported to be important for NGF to stimulate neurite outgrowth, supporting the point about structural remodelling and defects in end organ connectivity as underlying mechanisms for the observed phenotypic changes (Extended Fig. 13). There are also genes implicated in cell differentiation, cytoskeletal changes as well as cell organelles, such as lysosomes, endosomes, inflammasomes and processes that involved in cellular detoxification, such as ubiquitination and phase II metabolic enzymes. Please see complete list below for collaterally sprouted nociceptors as compared to the native innervation of the tibial territory in tables 3 and 4 below.

References:

Usoskin D, Furlan A, Islam S, Abdo H, Lönnerberg P, Lou D, Hjerling-Leffler J, Haeggström J, Kharchenko O, Kharchenko PV, Linnarsson S, Ernfors P. Unbiased classification of sensory neuron types by large-scale single-cell RNA sequencing. Nat Neurosci. 2015 Jan;18(1):145-53.

Table 3: Transcription analysis on IB4-negative DRG neurons innervating the tibial territory at 24-26 weeks post-SNI as compared to control mice:

**List of gene upregulated in SNI 24 wks over sham mice:**				
Number	Gene name	Protein	Putative function	Reference
1.	*Plat*	tissue plasminogen activator	Serine protease; Induced after nerve crush injury; tPA-plasmin signaling catalyses fibrin degradation and protects sciatic nerve axons upon injury	Akassoglou et al., JCB, 2000 PMID: **10831618**
2.	*Rin1*	Ras and Rab interactor 1	Effector for Ras-GTPase, cytoskeletal modulator in neurons, endosomal regulation<br>- activated downstream of NGF and EGF<br>- NGF-induced neurite outgrowth blocked by dominant-negative RIN or Rin knockdown<br>- In *C. elegans*, Rin1 regulates axonal pathfinding and neuronal cell migration	Spencer et a. JBC, 2002, PMID: **11877426** Shi et al. JBC, 2005 PMID: **16157584,** Doi et al., Development, 2013, PMID: **23900541**
3.	*Olfml3*	olfactomedin -like 3	Secreted glycoprotein; activated downstream of TGFß1 signaling in microglia; downregulated in ALS and increased expression improves functional parameters in ALS models; zebrafish homologs show axonal elongation defects	Neidert et al. Frontiers in Immunology, PMID: **30093905**
4.	*Tmem184c*	Trans-membrane protein 184c	Function unknown; its close family member Tmem184b localizes to recycling endosomes in axons; role in axonal degeneration; mutants show swellings with multivesicular bodies and abnormal sensory terminal morphology	Bhattacharya et al. J Neurosci. 2016
5.	*Tspan11*	tetraspanin 11	Function unknown, but other members of the family are implicated in neuroprotection; e.g., tetraspanin1 was found to be expressed in TrkA-expressing DRG neurons, increases the surface targeting of TrkA and is required for NGF-induced neurite outgrowth;	Ferrero et al. 2020 Cell Mol Life Sci., PMID: 31440771,<br><br>Masoudi et al. PLoS Genet. 2014, PMID: 25474638

			Tetraspanin 17 protects dopaminergic neurons, etc.	
6.	*Mcur1*	mitochondrial calcium uniporter regulator 1	Critically required for formation of mitochondrial calcium uniporter (MCU); promotes mitochondrial bioenergetics, which is dysregulated in neurodegeneration; implicated in neuroprotection	Tomar et al. Cell Reports, 2016 PMID: **27184846;** Jung et al. Front. Cell Develop. Biology 2020 PMID: **33392190**
7.	*Gstm2*	glutathione S-transferase mu 2	Phase II detoxification enzyme, involved in ROS detoxification, exerts a protective effect in response to metabolic stress, protects cells against autophagy and lysosome dysfunction<br>- Function in neurons unknown<br>- family member Gsta4 is associated with a highly significant increase in the survival of axotomized motoneurons and central neurons after brain injury	Ström et al. 2012, Neuromol. Med. PMID: **22160604**<br><br>Huenchuguala et al. 2014, Autophagy, PMID: **24434817**
8.	*Ppp2r1b*	protein phosphatase 2, regulatory subunit A, beta	Serine threonine protein phosphatase; neural function unknown	Uniprot.org; genecards.org
9.	*Knstrn*	kinetochore-localized astrin/SPAG5 binding	Small Kinetochore-Associated Protein, promotes stable microtubule-kinetochore attachments<br>-Neural function unknown, but human mutation associated with optic nerve atrophy	Sharfe et al. 2018<br><br>PMID: **29180244**
10.	*Ovol2*	ovo like zinc finger 2	Transcription factor in embryogenesis;<br>Required in neural tube development<br>Functions in adult nervous system unknown	Mackay et al. Developmental Biology, 2006, PMID: **16423343**
11.	*Msc*	Musculin	Basic helix-loop-helix transcription factor; functions in neurons unknown	Uniprot.org; genecards.org
12.	*Lrrc17*	leucine rich repeat containing 17	Function in neurons unknown	Uniprot.org; genecards.org
13.	ENSMUSG-	NA	NA	NA

	000000766 17			
14.	ENSMUS G- 000000808 93	NA	NA	
**List of gene <u>downregulated</u> in SNI 24 wks over sham mice:**				
Number	Gene name	Protein	Putative function	Reference
1.	*Lrguk*	leucine-rich repeats and guanylate kinase domain containing	Predicted to enable guanylate kinase activity, cytoskeletal regulator; function in neurons unknown	Okuda et al. 2017, FASEB J. PMID: **28003339**
2.	*Mrgpra3*	MAS-related GPR, member A3	Marker for the non-peptidergic (NP2) cluster of DRG nociceptors;<br>- Mrgpra3-expressing neurons implicated in nocifensive and pruriceptive responses;<br>- reduced in inflammatory pain models<br>- Mixed MrgprA3-MrgprC11 agonists induce hypersensitivity in bladder afferents	Usoskin et al., 2016 Nat. Neurosci, PMID: 25420068.<br><br>Grundy et al. J. Neurosci 2021 PMID: 33727332
3.	*Cela1*	chymotrypsin-like elastase family, member 1	Serine endopeptidase; neuronal function unknown; predicted to be a negative regulator of post-embryonic development and differentiation, predicted to be part of Wnt signaling	Uniprot.org, Ensembl databases

Table 4: Transcription analysis on IB4-positive neurons innervating the tibial territory at 24-26 weeks post-SNI as compared to control mice:

**List of gene <u>upregulated</u> in SNI 24 wks over sham mice:**				
Number	Gene name	Protein	Putative function	Reference
1.	*Eno1b*	enolase 1B	Glycolytic enzyme and can act as a surface receptor for plasminogen<br>- role in oxidative stress and protects against hypoxia<br>- novel protective role described in cerebral ischemia-induced neuronal injury, where it is upregulated post-injury, and also in stroke models	Jiang et al. ACS Chem Neurosci. 2019, PMID: **30943007;** Jiang et al. 2021 Neurosci. Lett
2.	ENSMUSG 000000808 93	NA	NA	

**List of gene <u>downregulated</u> in SNI 24 wks over sham mice:**				
Number	Gene name	Protein	Putative function	Reference
1.	*Clvs2*	clavesin 2	Neuron-specific; enriched in Clathrin-coated vesicles; Knockdown leads to alterations in lysosomal morphology	Katoh et al., JBC, 2009, PMID: **19651769**
2.	*Pstpip1*	proline-serine-threonine phosphatase-interacting protein 1	Role in inflammation, implicated in caspase and IL1 regulation cascade; functions in neurons unknown	Holzinger and Roth, Curr. Opin. Rheumatology, 2016, PMID: **27464597**
3.	*Rnf187*	ring finger protein 187	E3 ubiquitin-protein ligase, involved in protein uniquitination pathway, functions in neurons unknown	Uniprot.org
4.	*Gpr150*	G protein-coupled receptor 150	Orphan GPCR; suggested to be related to Oxytocin-Vasopressin receptor family, function unknown	Gloriam et al. 2005, Biochim Biophys Acta PMID: **15777626.**
