# Supplementary material for: Neuropathic pain caused by miswiring and abnormal end organ targeting
Source: Nature. 2022 May 25;606(7912):137–45. doi: 10.1038/s41586-022-04777-z (PMC9159955; doi:10.1038/s41586-022-04777-z)
Supplement: Supplementary file 2 — Reporting Summary [file 41586_2022_4777_MOESM2_ESM.pdf]

nature research

Corresponding author(s): Prof. Dr. Rohini Kuner

Last updated by author(s): Mar 3, 2022

Reporting Summary

Nature Research wishes to improve the reproducibility of the work that we publish. This form provides structure for consistency and transparency in reporting. For further information on Nature Research policies, see our Editorial Policies and the Editorial Policy Checklist.

Statistics

For all statistical analyses, confirm that the following items are present in the figure legend, table legend, main text, or Methods section.

n/a	Confirmed	
☐	☒	The exact sample size (*n*) for each experimental group/condition, given as a discrete number and unit of measurement
☐	☒	A statement on whether measurements were taken from distinct samples or whether the same sample was measured repeatedly
☐	☒	The statistical test(s) used AND whether they are one- or two-sided<br>*Only common tests should be described solely by name; describe more complex techniques in the Methods section.*
☒	☐	A description of all covariates tested
☐	☒	A description of any assumptions or corrections, such as tests of normality and adjustment for multiple comparisons
☐	☒	A full description of the statistical parameters including central tendency (e.g. means) or other basic estimates (e.g. regression coefficient) AND variation (e.g. standard deviation) or associated estimates of uncertainty (e.g. confidence intervals)
☐	☒	For null hypothesis testing, the test statistic (e.g. *F*, *t*, *r*) with confidence intervals, effect sizes, degrees of freedom and *P* value noted<br>*Give P values as exact values whenever suitable.*
☒	☐	For Bayesian analysis, information on the choice of priors and Markov chain Monte Carlo settings
☒	☐	For hierarchical and complex designs, identification of the appropriate level for tests and full reporting of outcomes
☒	☐	Estimates of effect sizes (e.g. Cohen's *d*, Pearson's *r*), indicating how they were calculated

Our web collection on statistics for biologists contains articles on many of the points above.

Software and code

Policy information about availability of computer code

Data collection	Imspector Pro (LaVision); Leica Application Suite X (LAS X); several custom-written softwares for EM image acquisition - available at Gitlab (https://gitlab.mpcdf.mpg.de/connectomics/emacquisitionmacro.git).
Data analysis	Software used for analysis include Sigmaplot, Image J, Microsoft Excel, ANYmaze, custom-written codes for image analysis of multiphoton imaging data - available at Github (https://github.com/zheng-tklab/pns_2photon_longitudinal-3Ddata-analysis).

For manuscripts utilizing custom algorithms or software that are central to the research but not yet described in published literature, software must be made available to editors and reviewers. We strongly encourage code deposition in a community repository (e.g. GitHub). See the Nature Research guidelines for submitting code & software for further information.

Data

Policy information about availability of data

All manuscripts must include a data availability statement. This statement should provide the following information, where applicable:

- Accession codes, unique identifiers, or web links for publicly available datasets
- A list of figures that have associated raw data
- A description of any restrictions on data availability

All of the raw data for behavior analyses, electrophysiology and immunohistochemistry analyses are provided in source data file and in the figures. RNA sequencing data are available on the European Nucleotide Archive (https://www.ebi.ac.uk/ena) under the accession number PRJEB50184. The raw data for multiphoton imaging and EM analyses are available upon request.

Field-specific reporting

Please select the one below that is the best fit for your research. If you are not sure, read the appropriate sections before making your selection.

☒ Life sciences ☐ Behavioural & social sciences ☐ Ecological, evolutionary & environmental sciences

For a reference copy of the document with all sections, see nature.com/documents/nr-reporting-summary-flat.pdf

Life sciences study design

All studies must disclose on these points even when the disclosure is negative.

Sample size	Our sample sizes are also similar to those reported in previous publications. In previous studies we have determined the sample size using G-power analyses and therefore have a very clear set of what sample size is required for the behavioral and histochemical data reported.
Data exclusions	A mouse showing autotomy following spared nerve injury (SNI) was excluded from the analysis as it damages the imaging tissue volume.
Replication	All experiments were successfully replicated multiple times with several animals. The precise animal numbers are given in the figure legends.
Randomization	Groups were randomized and mice were allocated to experimental groups by a researcher different from the experimentor
Blinding	Experimentor was blinded to the identity of mice being analyzed in behavioral tests

Reporting for specific materials, systems and methods

We require information from authors about some types of materials, experimental systems and methods used in many studies. Here, indicate whether each material, system or method listed is relevant to your study. If you are not sure if a list item applies to your research, read the appropriate section before selecting a response.

Materials & experimental systems

n/a	Involved in the study	
☐	☒	Antibodies
☒	☐	Eukaryotic cell lines
☒	☐	Palaeontology and archaeology
☐	☒	Animals and other organisms
☒	☐	Human research participants
☒	☐	Clinical data
☒	☐	Dual use research of concern

Methods

n/a	Involved in the study	
☒	☐	ChIP-seq
☒	☐	Flow cytometry
☒	☐	MRI-based neuroimaging

Antibodies

Antibodies used

Primary antibodies:
Anti-beta-tubulin III (T2200, sigma; 1:500, raised in Rabbit), anti-NF200 (CH23015, Neuromics; 1:200, raised in Chicken), anti-CGRP (24112, Immunostar; 1:200, raised in Rabbit), anti-Substance P (GP14103 Neuromics; 1:200, raised in Ginuea Pig), anti-S100 (Z0311, Dako; 1:200), anti-TROMA-I (Krt8, DSHB; 1:1000), anti-TH (SO25000, Neuromics; 1:200) and biotinylated Isolectin B4, IB4 (B-1205, Vector, 1:200), anti Gr-1(Mouse Ly-6G/ly-6C)(MAB 1037, R&D systems; 1.500), anti-CD8a (14-0808-82, Thermo Fisher; 1:200), anti-CD4 (14-9766-82, Thermo Fisher; 1:100)

Secondary antibodies:
Donkey anti-Rabbit IgG, Alexa 488 conjugated (Cat # A32790)
Donkey anti-Rabbit IgG, Alexa 594 conjugated (Cat # A32754)
Donkey anti-Rabbit IgG, Alexa 647 conjugated (Cat # A32787)
Donkey anti-Rat IgG, Alexa 647 conjugated (Cat # A48272)
Donkey anti-Rat IgG, Alexa 594 conjugated (Cat # A48271)
Donkey anti-Rat IgG, Alexa 488 conjugated (Cat # A48269)
Goat anti-Guinea pig IgG, Alexa 647 conjugated (Cat # A-21450)

All of these above secondary antibodies were purchased from Thermo Fisher Scientific.

Validation

All the primary antibodies were used in non-living tissue for immunohistochemistry (IHC). These antibodies are extensively used for IHC purpose by the scientific community.

References:

Neubarth NL, Emanuel AJ, Liu Y, Springel MW, Handler A, Zhang Q, Lehnert BP, Guo C, Orefice LL, Abdelaziz A, DeLisle MM, Iskols M, Rhyins J, Kim SJ, Cattel SJ, Regehr W, Harvey CD, Drugowitsch J, Ginty DD. Meissner corpuscles and their spatially intermingled afferents underlie gentle touch perception. Science. 2020 Jun 19;368(6497):eabb2751.

Selvaraj D, Gangadharan V, Michalski CW, Kurejova M, Stösser S, Srivastava K, Schweizerhof M, Waltenberger J, Ferrara N, Heppenstall P, Shibuya M, Augustin HG, Kuner R. A Functional Role for VEGFR1 Expressed in Peripheral Sensory Neurons in Cancer Pain. Cancer Cell. 2015 Jun 8;27(6):780-96.

Tong Liu, Temugin Berta, Zhen-Zhong Xu,Chul-Kyu Park,Ling Zhang, Ning Lü, Qin Liu, Yang Liu, Yong-Jing Gao, Yen-Chin Liu, Qiufu Ma, Xinzhong Dong, and Ru-Rong Ji. (2012). TLR3 deficiency impairs spinal cord synaptic transmission, central sensitization, and pruritus in mice. J Clin Invest. 122(6): 2195–2207.

Animals and other organisms

Policy information about studies involving animals; ARRIVE guidelines recommended for reporting animal research

Laboratory animals	Adult (8-56 weeks) C57Bl6 male/female mice (25 - 30 g) of wild-type, Thy1-YFP, SNS-mGFP, SNS-tdTomato, SNS-iDTR were used in this study.Mice were housed in groups of 2–3 per cage (in ventilation unit) with food and water ad libitum on a 12 h light / 12 h dark cycle. Room temparature and humidity were ranging from 20-23 °C and 40-60%. A detailed description of transgenic lines are available in supplementary information.
Wild animals	No wild animals were used in this study
Field-collected samples	No field collected samples were used this study
Ethics oversight	All of the animal experiments were conducted according to the ethical guidelines of 'Protection of Animals Act' under supervision of the 'Animal Welfare Officers' of Heidelberg University and were approved by the local governing body named 'Regierungspräsidium Karlsruhe: Abteilung 3 - Landwirtschaft, Ländlicher Raum, Veterinär- und Lebensmittelwesen', Germany (Approval numbers: G-206/11 and G-177/17). ARRIVE guidelines were followed.

Note that full information on the approval of the study protocol must also be provided in the manuscript.
